# Extracellular vesicles for next-gen therapeutics and drug delivery

**DOI:** 10.1186/s43556-026-00571-9

**Published:** 2026-09-09

**Authors:** Nawaz Khan, Zeynab Ahmadova, Yujie Shi, Tayyab Shafiq, Yujie Liang, Li Duan

**Affiliations:** 1 Department of Orthopedics, Guangdong Artificial Intelligence Biomedical Innovation Platform, Shenzhen Second People’s Hospital, the First Affiliated Hospital of Shenzhen University, Shenzhen, Guangdong 518035 China; 2 Medical Innovation Technology Transformation Center of Shenzhen Second People’s Hospital, the First Affiliated Hospital of Shenzhen University, Shenzhen, Guangdong 518035 China; 3 School of Medicine, Shenzhen University, Shenzhen, Guangdong 518060 China; 4 College of Rehabilitation Medicine, Jining Medical University, Jining, Shandong 272067 China

**Keywords:** Extracellular vesicles, Exosomes, Drug delivery, Vaccine, Immunotherapy

## Abstract

Extracellular vesicles (EVs) are membrane-bound, nano-sized particles released by diverse cell types. They serve as key mediators of intercellular communication by transporting a broad repertoire of proteins, lipids, nucleic acids, and metabolites to recipient cells within a protective lipid bilayer. Owing to their natural origin, intrinsic stability, low immunogenicity, and ability to cross biological barriers, EVs are highly attractive candidates for next-generation therapeutics and drug delivery platforms. In this review, we summarize the biology of EVs and highlight the features critical for their application in therapeutic delivery. We trace their multiple origins, ranging from mammalian and plant cells to bacteria, underscoring their ubiquity across biological systems. We then analyze the advantages of EVs as next-generation delivery vehicles. From a practical standpoint, we examine current strategies for EV isolation, purification, characterization, and engineering. Importantly, we showcase several therapeutic applications of EVs for intractable diseases that are refractory to conventional approaches, such as cancer and immune disorders. We also discuss the major challenges on the path to clinical translation, particularly scalable and standardized production, as well as safety and immunogenicity. To fully realize the clinical potential of EV-based therapies, future research should prioritize the development of robust manufacturing protocols and comprehensive safety evaluations.

## Introduction

Extracellular vesicles (EVs) are nanoscale, membrane-bound particles released by all cell types into the extracellular space where they serve as critical mediators of intercellular communication [1]. Surrounded by a lipid bilayer, these vesicles encapsulate a wide spectrum of bioactive molecules such as proteins, lipids, nucleic acids, and metabolites that often reflect the molecular status of their parental cells [2]. Unlike freely circulating biomolecules, EV-associated cargo is protected from enzymatic degradation and also ensures the precise delivery of functional biomolecules to recipient cells [3]. These natural transportation properties enable EVs to reach diverse tissues, where they regulate numerous physiological processes (*e.g*., immune modulation and tissue repair) and pathological processes (*e.g*., aberrant inflammatory signaling and disease progression). Their ability to naturally traverse biological barriers and deliver intact cargo has positioned them as promising next-generation (next-gen) platforms for therapeutic interventions and targeted drug delivery [4].

In recent years, EVs have attracted considerable interest as biologically compatible drug delivery systems. These features confer distinct advantages over synthetic nanocarriers and viral vectors, including minimal immunogenicity, enhanced stability, and efficient cellular uptake, which together enable the transport of diverse therapeutic cargoes [5]. One of their defining features is their innate capacity for targeted delivery. Specific surface components such as integrins, tetraspanins, lipids, and glycan structures mediate selective interactions with receptors on recipient cells and enable tissue-specific internalization while minimizing off-target distribution [6]. This property allows EVs to cross physical barriers (*e.g*., vascular endothelium [7], blood brain barrier (BBB)) and overcome the restrictive nature of the dense tumor microenvironment [8]. These delivery properties position them as remarkable tools for the treatment of neurological diseases and brain tumors, where conventional drugs show limited penetration [8, 9]. Moreover, engineering advances now enable modification of EV targeting, loading efficiency, and release kinetics, which further expand their therapeutic potentials [10].

In addition, EVs have emerged as powerful candidates for vaccine development. EVs derived from antigen-presenting cells like dendritic cells (DCs) and macrophages, or even from tumor cells, intrinsically carry immunostimulatory components [11]. Their natural ability to activate immune responses without additional adjuvants makes them ideal for therapeutic vaccine design [12]. Moreover, by loading EVs with specific antigen cargo, it is possible to generate potent vaccine formulations capable of efficient lymphoid tissue targeting and evasion of premature immune clearance [13]. On the other hand, a rapidly developing frontier in EV research lies in cancer immunotherapy [14]. Although existing approaches, such as chimeric antigen receptor (CAR) T cell therapy and immune checkpoint inhibitors, have produced notable clinical outcomes [15], they are frequently associated with serious adverse effects such as cytokine release syndrome and organ toxicity [16]. In this scenario, EVs offer a safer and more controlled alternative by delivering tumor-associated antigens or immunomodulatory molecules directly to immune cells, thereby promoting robust antitumor responses while mitigating risk of immune overactivation [17].

Despite EVs' remarkable potential, several complications hinder their clinical translation. These limitations include difficulties in efficient cargo loading [18], rapid clearance from the circulation [19], and challenges associated with scalable and reproducible production [20]. However, recent advances in EV engineering, together with improvements in manufacturing, purification, and standardization strategies, are gradually helping to address these barriers. Overall, the unique biological functions and engineering adaptability of EVs position them at the forefront of next-gen therapeutics. Their natural compatibility, capacity to transport complex biomolecules, and ability to cross critical physiological barriers provide significant advantages for applications covering oncology, autoimmune diseases, and regenerative medicine [21]. As technological innovations address current production and engineering challenges, EVs are poised to become a transformative tool in future therapeutic strategies.

This review focuses specifically on EVs as next-gen therapeutic and drug-delivery platforms rather than providing only a general overview of EV biology. Therefore, foundational aspects such as EV biogenesis, classification, functional roles, surface markers, isolation, purification, and characterization are discussed mainly in relation to their relevance for therapeutic design, cargo loading, targeting, quality control, and clinical translation. The review also highlights the major sources of EVs and current engineering strategies, followed by a comprehensive discussion of their therapeutic applications, with particular emphasis on vaccine development, cancer immunotherapy, and advanced drug delivery systems, which represent some of the most promising and rapidly evolving areas of EV-based medicine. By linking EV biology with therapeutic engineering and clinical applications, this review aims to highlight the unique advantages, current challenges, and future prospects of EV-based therapeutics.

## Biology of EVs

### EV biogenesis

**Fig. 1:**
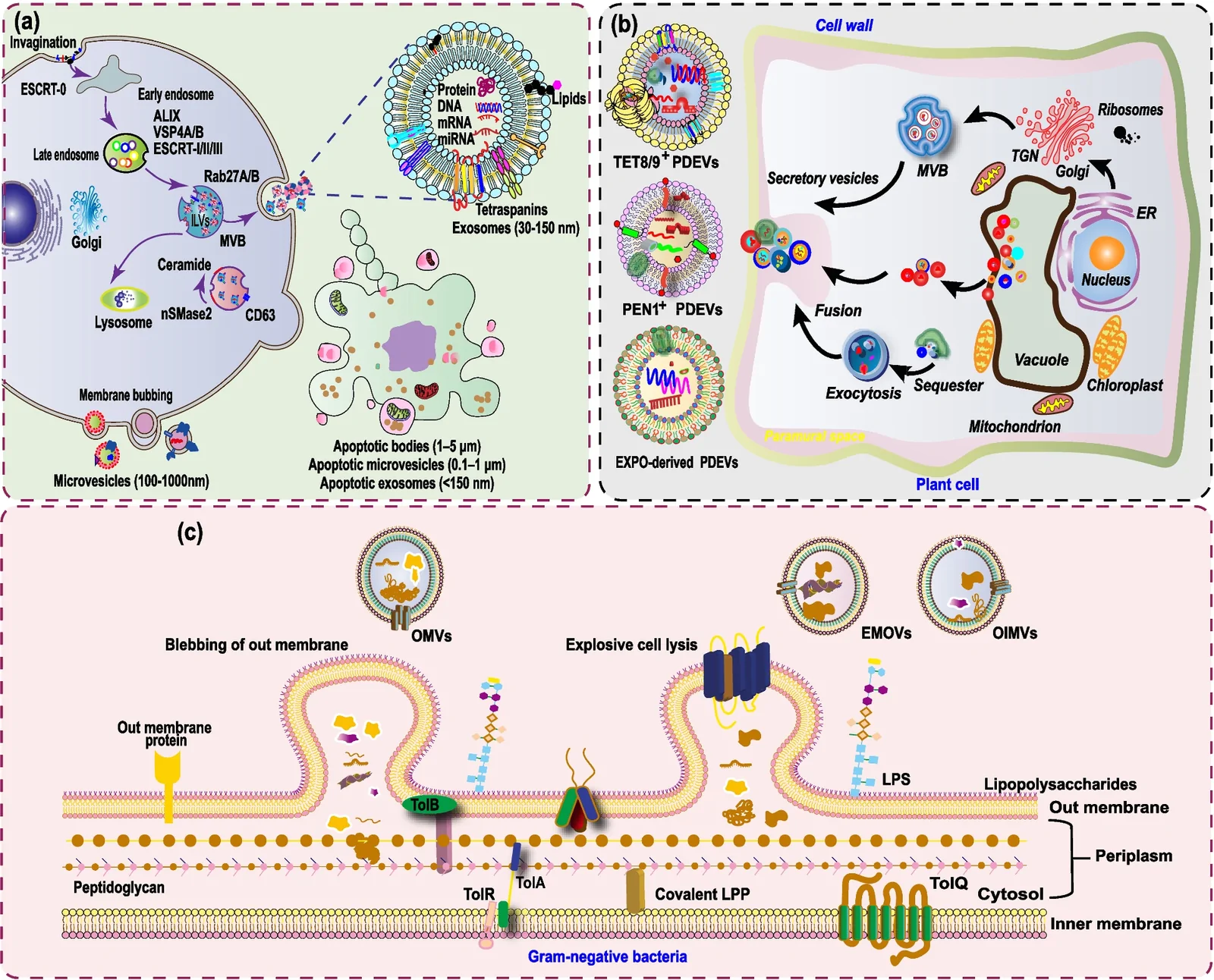
Biogenesis pathways of EVs in mammals, plants, and bacteria. **a** Mammalian EVs biogenesis: EVs are generated through different pathways. Exosomes (30 −150 nm) are formed via inward budding of the early endosomal membrane to produce MVBs and involve key regulators such as ESCRT (endosomal sorting complex required for transport)−0/I/II/III, ALIX (ALG-2 interacting protein X), VSP4A/B (vacuolar protein sorting-associated protein 4A/B), ceramide, nSMase2 (neutral sphingomyelinase 2), Rab27A/B (Ras-associated binding proteins), and tetraspanins (*e.g*., CD63). Fusion of MVBs with the plasma membrane releases exosomes. Microvesicles (100 −1000 nm) arise through outward budding of the plasma membrane. Apoptotic bodies (1–5 μm) are generated during programmed cell death through membrane blebbing. **b** PDEV biogenesis: PDEVs originate mainly from MVBs and TGN (trans-Golgi network). Vesicles fuse with the plasma membrane to release several subtypes, including TET8⁺ PDEVs (Tetraspanin 8-positive PDEVs), PEN1⁺ PDEVs (Penetration 1-positive PDEVs), and EXPO-derived PDEVs (Exocyst-positive organelle-derived PDEVs). Plant cells contain distinct compartments, such as the paramural space and vacuole, that participate in EV cargo sequestration and release. PDEV cargo includes proteins, lipids, metabolites, DNA, mRNA, and small RNAs. **c** Bacteria-EVs (OMVs) biogenesis: Gram-negative bacteria generate OMVs through outward blebbing of the outer membrane, and EMVs (explosive membrane vesicles) through cell-lysis-driven membrane fragmentation. They may also form OIMVs (outer-inner membrane vesicles) that encapsulate both membranes. OMVs include components such as LPS, outer membrane proteins, peptidoglycan, and cytosolic cargo. Membrane dynamics during vesiculation are regulated by the Tol–Pal system (TolQ/TolR/TolA/PAL) and covalent LPP (murein lipoprotein) linkages

Exosome biogenesis is a multi-step process that originates from the cell's endocytic pathway [22]. It begins with the internalization of plasma membrane (PM) segments to form early endosomes that function as a primary sorting station [23]. These organelles mature into late endosomes and subsequently into multivesicular bodies (MVBs). These MVBs are characterized by the presence of numerous intraluminal vesicles (ILVs) within their lumen [24]. The ILVs are generated by the inward budding of the MVB's own membrane. The MVB can then be directed toward either lysosomal degradation or PM fusion for exosome secretion. Upon fusion with the PM, the ILVs are released into the extracellular space, and at this point, they are termed exosomes (Fig. 1) [25].

The formation and selective loading of ILVs are mainly regulated by both endosomal sorting complexes required for transport (ESCRT)-dependent and ESCRT-independent pathways [25, 26]. In the ESCRT-dependent pathway, ESCRT complexes and the ATPase Vps4 orchestrate a coordinated cascade of cargo recognition, membrane budding, scission, and recycling of the sorting machinery [27–30]. This series of events is frequently organized within the tetraspanin-enriched microdomains. These microdomains act as scaffolding platforms for both cargo and the associated machinery [31]. In parallel, ESCRT-independent mechanisms–such as ceramide-mediated lipid remodeling-also promote membrane curvature and ILV budding [32]. In addition, specific cargo-loading systems direct the selective incorporation of cytosolic proteins and microRNAs (miRNAs) into ILVs through motif or RNA-binding-protein-dependent recognition [33, 34]. Following ILV formation, MVB trafficking and secretion are controlled by cytoskeletal motors, Rab GTPases, tethering factors, and soluble N-ethylmaleimide-sensitive factor attachment protein receptor (SNARE)-mediated fusion with the PM [35–39]. These regulatory steps are functionally consequential, as they dictate whether MVB contents are committed to lysosomal degradation or secreted as exosomes. Therefore, exosome biogenesis is not merely a fundamental biological process but also a key determinant of exosomal cargo composition, surface features, therapeutic loading capacity, and potential clinical application [40].

Microvesicles (MVs), also known as ectosomes, represent another class of EVs. MVs are generated by a distinct mechanism involving the direct outward budding and scission of the PM [41]. MV biogenesis is initiated by localized changes in the PM, including lipid rearrangement, membrane bending, phosphatidylserine externalization, and cytoskeletal remodeling [42–44]. These events are coordinated by membrane-associated proteins, including tetraspanins such as CD9 and CD81, as well as actomyosin contractility regulated by the Ras homolog gene family member A (RhoA)/Rho associated protein kinase (ROCK) pathway, which promotes PM blebbing and vesicle release [45, 46]. In some circumstances, ESCRT machinery components, such as TSG-101, are also recruited to the PM to participate in the final scission event, representing a non-canonical role of this typically endosomal machinery [47]. Furthermore, the loading of cargo into budding MVs is a selective process influenced by lipid rafts, membrane microdomains, and specific protein–RNA sorting mechanisms [48–50]. On the other hand, MVs are not a monolithic population but a diverse group of vesicle subtypes that include oncosomes, migrasomes, and ciliary MVs, which differ in size, origin, cargo composition, and biological function [51–53].

Apoptotic bodies (ApoBDs) are membrane-enclosed vesicles that are generated during apoptotic cell fragmentation and play an important role in the efficient clearance of dying cells by phagocytes [54, 55]. Their formation is initiated by apoptotic volume decrease, plasma membrane blebbing, actomyosin contraction, and the development of membrane extensions such as apoptopodia, which collectively promote the packaging and separation of cellular components into discrete vesicles [56, 57]. By sequestering potentially toxic intracellular contents into these sealed vesicles**,** this process prevents the uncontrolled release of intracellular content and thereby suppresses inflammatory responses.

Despite significant advances in understanding the biogenesis of EVs- including the identification of major pathways governing their formation- several challenges remain. In particular, the precise molecular mechanisms that underline vesicle heterogeneity, cargo selectivity, surface marker composition, and functional specialization are still not fully understood. These knowledge gaps are particularly critical, as EV biogenesis directly determines the biological properties, targeting capabilities, and therapeutic potential of different EV subtypes from mammalian, plant, and bacterial systems (Fig. 1).

### The composition of EVs

The composition of EVs is highly diverse and depends on their cellular origin, physiological state, and biogenesis pathway. EVs contain a complex mixture of proteins, lipids, nucleic acids, metabolites [58–61], and other bioactive molecules that determine their biological activity and therapeutic potential. Their internal cargo may include enzymes, cytokines, growth factors, messenger RNAs (mRNAs), miRNAs, and other non-coding RNAs. Therefore, EV composition is a major determinant of their ability to regulate gene expression [62], modulate immune responses, and promote tissue repair [63].

The EV surface is a biologically active interface that dictates its interactions with the surrounding environment, including target cell recognition, binding, and uptake. This interface is decorated with a diverse collection of lipids, proteins, and glycans that function as molecular "addresses" and mediate interactions with recipient cells [25]. Importantly, surface composition varies according to the parental cell type and biogenesis pathway, such as endosomal sorting for exosomes or direct budding from the PM for MVs [64, 65]. This feature is highly relevant for therapeutic design because it influences circulation behavior, immune recognition, tissue tropism, and delivery efficiency.

Several surface proteins serve as both EV identifiers and functional mediators. Tetraspanins, such as CD9, CD63, and CD81, are nearly ubiquitous on exosomes and play central roles in organizing membrane microdomains, cargo sorting, and cellular interactions [31]. Similarly, integrins found on the EV surface contribute to cellular adhesion and signaling [66]. Additionally, major histocompatibility complex (MHC) class I and II molecules present on the surface reflect the cellular origin of the EVs and are critical for antigen presentation [67]. Furthermore, EVs retain parent-cell-specific surface signatures. For example, tumor-derived EVs (TD-EVs) may display programmed death receptor 1 (PD-1) or human epidermal growth factor receptor 2 (HER2) [68], whereas stem cell-derived EVs may express regenerative markers such as CD105 [69], and endothelial EVs may carry adhesion molecules such as intercellular adhesion molecule-1 (ICAM-1), which are involved in vascular homeostasis and inflammation [70]. These markers are important for EV characterization and may also help predict therapeutic efficacy, targeting specificity, and in vivo safety profiles.

The lipid and glycan composition of EVs further shapes their stability, signaling activity, immune interactions, and clearance. EV membranes contain phospholipids, phosphatidylserine, sphingolipids, and cholesterol, which contribute to vesicle stability and membrane organization [71, 72]. The EV glycocalyx, composed of glycoproteins and glycans inherited from the parental cell regulates adhesion, immune recognition, biodistribution, and clearance [73, 74]. Specific glycan patterns, such as elevated sialylation [75], or high-mannose N-glycan [76], may influence immune evasion, metastasis, or liver clearance.

EV composition may also change after vesicles enter biological fluids. In circulation, EVs can acquire proteins, lipoproteins, RNAs, and other molecules from the surrounding environment, forming a biomolecular corona [77–79]. This acquired corona can modify the biological identity of EVs and influence circulation time, cellular uptake, immune interactions, and biodistribution [80]. Thus, a comprehensive understanding of both native EV composition and acquired surface modifications is essential for improving EV-based therapeutic delivery, quality control, and clinical translation.

### Key features supporting therapeutic delivery

EVs play crucial roles in physiological communication and homeostasis. However, their complete mechanisms of action in vivo are still being explored [1, 81]. Their inherent ability to transfer bioactive molecules between cells enables them to regulate diverse biological processes and has generated significant interest in therapeutic development. For instance, human placental stromal cell-derived EVs can promote angiogenesis, enhance skin regeneration, and modulate immune responses [82]. Additionally, cancer cell-derived EVs can interact with lipoproteins to influence their uptake and biodistribution in the body, illustrating their functional complexity and adaptability [83, 84].

One of the key features supporting EV-based therapeutic delivery is their ability to protect molecular cargo. Their lipid bilayer provides an efficient barrier that shields sensitive biomolecules such as RNA, growth factors, cytokines, etc*.*, from environmental stress and enzymatic degradation [85]. This protective ability ensures the stability and bioactivity of the encapsulated materials during transport in EVs. Furthermore, EVs also display exceptional physical robustness. They can tolerate repeated freeze–thaw cycles and processes like lyophilization while maintaining structural integrity, which makes them suitable for long-term storage and experimental applications [86, 87].

In addition, EVs also demonstrate a remarkable ability to traverse biological barriers, such as the vascular endothelium and the BBB [9], which allows them to deliver cargo to otherwise inaccessible tissues. Although the precise mechanisms governing this process still remain under investigation, it is generally believed that this ability may involve receptor-mediated endocytosis or transcytosis. Another essential natural function of EVs is their ability to evade immune recognition. As EVs are composed of endogenous lipids and proteins from their source cells, autologous EVs typically do not elicit strong immune responses, supporting their development as personalized and cell-free therapies [88, 89]. However, the immunogenicity of EVs may vary depending on factors such as their molecular composition, cellular origin, and size.

Furthermore, the targeted interaction with recipient cells represents another vital aspect of EV function. Specific surface molecules, including glycans, integrins, tetraspanins, and adhesion proteins, mediate the recognition and selective binding of EVs to their target cells [90, 91]. These molecules allow EVs to deliver their cargo with high precision and specificity. Additionally, this natural targeting capacity of EVs can be enhanced through biological or chemical engineering approaches. However, these modifications must preserve the natural integrity and functionality of the EVs [92]. Once bound to target cells, EVs can modulate cellular physiology through receptor-mediated signaling [93] and local paracrine effects, in addition to gaining intracellular access through endocytosis, phagocytosis, and macropinocytosis [85], as well as direct membrane fusion that allows the rapid delivery of cargo into the cytoplasm [94, 95]. Thus, EVs possess diverse biological functions, including cargo protection, immune compatibility, barrier crossing, cellular targeting, and functional delivery, making them attractive platforms for next-gen therapeutics and drug delivery.

## Sources of EVs as therapeutics

EVs play a fundamental role in intercellular communication and are secreted by various cell types. They act as messengers that coordinate complex physiological processes and maintain tissue homeostasis [96]. In a physiological setting, these naturally secreted EVs can be taken up by nearby cells or may travel through the circulation to reach and act on target cells within distant tissues and organs. Under physiological conditions, EVs regulate the local microenvironment and maintain homeostasis [97]. In pathological settings, however, such as tumors, EVs released by cancer cells can modulate the surrounding environment to promote immunosuppression and drug resistance. This dual capacity underscores their potent and versatile biological activity [98]. Given the complexity of these endogenous systems**,** translating such in vivo phenomena directly into therapies is challenging. To overcome this challenge, researchers have focused on isolating natural EVs from more controlled sources for therapeutic development and use. These sources mainly include cell culture supernatants of mammalian cells, biological fluids, plant tissues, and bacteria [21]. In this section, the therapeutically relevant characteristics of EVs derived from mammalian cells, plants, and bacteria are discussed (Table 1). Their key differences are comparatively summarized, including in size and structure, membrane composition, cargo characteristics, manufacturability, safety, engineering flexibility, and clinical readiness.

**Table 1:** Comparative characteristics of mammalian cell, plant, and bacteria-derived EVs

Feature	Mammalian cell-derived EVs	Plant-derived EVs	Bacteria-derived EVs	Reference
Representative sources	MSCs, iPSCs, immune cells, cancer cells, and blood cells	Edible and medicinal plants, fruits, vegetables, roots, seeds, and leaves	Gram-negative, Gram-positive	[99–105]
Size and structure	Lipid-bilayer vesicles comprising exosomes, MVs, and ApoBDs. Exosomes are typically 30–150 nm in diameter, MVs range from approximately 100 nm to 1 μm, and ApoBDs generally range from 100 nm to 5 μm	Lipid-bilayer vesicles generally ranging from approximately 50–1000 nm; exosome are commonly 50–150 nm	Nanosized, spherical lipid-bilayer vesicles; approximately 20–250 nm, although larger populations may occur	[104, 106–108]
Membrane composition	Rich in phospholipids, cholesterol, sphingomyelin, ceramides, glycolipids, and source-specific membrane proteins; composition varies among EVs subtypes	Contain plant-specific phospholipids, sphingolipids, phosphatidic acid, and glycosylinositolphosphoceramides; generally, lack cholesterol-rich membrane domains characteristic of mammalian EVs	Contain bacterial phospholipids such as phosphatidylethanolamine, phosphatidylglycerol, phosphatidylinositol, and cardiolipin; Gram-negative EVs additionally contain LPS and outer-membrane proteins	[109–113]
Cargo characteristics	Carrying proteins, lipids, metabolites, mRNAs, miRNAs, and other non-coding RNAs that reflect the parental-cell phenotype and physiological state	Carrying proteins, lipids, plant miRNAs, defense-related RNAs, antimicrobial and hydrolytic enzymes, antioxidant enzymes, and plant secondary metabolites	Carrying proteins, enzymes, metabolites, DNA, RNA, membrane components, and immunologically active molecules; pathogenic EVs may also contain toxins, virulence factors, and antibiotic-resistance enzymes	[114–122]
Manufacturability	Production requires controlled cell culture, donor qualification, and scalable purification; yield and consistency may be affected by cell senescence, phenotype, passage number, and culture conditions	Plants are abundant, renewable, inexpensive, and do not require mammalian cell culture; however, cell-wall disruption and source-dependent variability complicate standardization	Rapid bacterial growth, mature fermentation methods, high production yield, and ease of genetic manipulation support scalable production; composition varies with strain, growth phase, and culture conditions	[123–125]
Safety	Generally biocompatible, but safety depends on parental-cell identity, genetic stability, purity, biodistribution, and biological potency; cancer-cell EVs require particular caution	Generally considered to have low toxicity and immunogenicity and minimal risk of transmitting human pathogens; contaminants such as pesticides, heavy metals, microbes, and plant allergens must be controlled	Safety is strongly source dependent; LPS, peptidoglycan, toxins, and virulence factors may induce inflammation or cytotoxicity. Probiotic, attenuated, or genetically detoxified strains provide safer alternatives	[21, 126, 127]
Engineering flexibility	Can be modified through parental-cell engineering, surface functionalization, electroporation, sonication, incubation, and ligand display; suitable for proteins, nucleic acids, and small-molecule delivery	Can be loaded through incubation, electroporation, sonication, and extrusion and functionalized with targeting ligands; their natural stability supports oral delivery	Highly amenable to genetic engineering, recombinant protein display, synthetic biology, CRISPR-based detoxification, electroporation, sonication, incubation, and surface modification	[124, 128–130]
Clinical readiness	Among naturally derived EVs, the MSC-EV category is the most clinically advanced; several mammalian MSC-EV products and DC-EV vaccines have entered clinical trials, although no broadly approved therapeutic EV product is yet available	Predominantly supported by in vitro and in vivo studies; limited early clinical investigation has begun, but GMP manufacturing and regulatory classification remain unresolved	OMV-based vaccines such as Bexsero® are clinically approved, demonstrating translational feasibility; most engineered bacterial EV systems for drug delivery and regenerative medicine remain preclinical	[131, 132]
Principal strengths	High biological compatibility, source-specific targeting, diverse cargo, and strong therapeutic relevance	Low-cost production, oral stability, plant-specific bioactive cargo, and favorable safety profile	High yield, rapid production, strong immunostimulatory capacity, and exceptional genetic engineerability	[21, 123, 133, 134]
Main limitations	Expensive production, donor variability, limited yield, complex quality control, and possible source-related safety concerns	Heterogeneous composition, lack of universal markers, variable isolation efficiency, and insufficient manufacturing standardization	Endotoxin and virulence-related toxicity, strong immune activation, source-dependent cargo, and limited long-term safety data	[20, 21, 127, 135, 136]

### Mammalian cell-derived EVs

Mammalian cell-derived EVs represent the most extensively investigated class of therapeutic vesicles because of their close biological compatibility with human tissues and their ability to reproduce many functional properties of their parental cells [137]. Their ability to transfer functional molecules between cells enables them to regulate physiological and pathological processes and has generated considerable interest in their development as therapeutic agents, biomarkers, and drug-delivery platforms. Compared with many synthetic nanocarriers, mammalian EVs possess high biocompatibility, protect their cargo from degradation, and traverse biological barriers [138]. Their size varies according to the biogenesis pathway, with exosomes typically ranging from 30–150 nm, MVs from 100 nm to 1 μm, and ApoBDs from 100 nm to 5 μm in diameter [106]. These vesicles are enclosed by lipid-bilayer membranes and may display spherical or cup-shaped morphologies. The membrane lipid composition of EVs is commonly enriched in cholesterol, sphingomyelin, glycosphingolipids, and phosphatidylserine [109]. Moreover, gangliosides, including GM1, GM2, GM3, and GD3, are prominent glycolipid components of mammalian EV membranes [139]. Ceramide contributes to membrane curvature and participates in ESCRT-independent intraluminal vesicle formation [32]. Mammalian EVs may additionally carry bioactive lipids that regulate inflammatory, metabolic, and disease-associated signaling pathways in recipient cells [114].

Their protein composition comprises membrane proteins and internal soluble proteins. Tetraspanins such as CD9, CD63, CD81, and CD82 organize membrane microdomains and are frequently used as EV-associated markers [138, 140]. Integrins present on EV surfaces contribute to adhesion, cellular recognition, and tissue-specific targeting [141]. Proteins associated with EV biogenesis, including ALIX and TSG101, are frequently detected in mammalian EV preparations [142]. In addition, their luminal cargo can contain cytosolic proteins, metabolic enzymes, signaling molecules, cytokines, and heat-shock proteins such as heat shock protein (HSP)70 and HSP90 [143]. These EVs also transport diverse nucleic acids. Both single- and double-strand DNA have been detected in EV preparations [115]. They are particularly enriched in RNA species, including mRNAs, miRNAs, long non-coding RNAs, circular RNAs, and small nucleolar RNAs.

Beyond their molecular composition, mammalian EVs arise from diverse cellular and biological sources, including stem cells, immune cells, and cancer cells, as well as blood-derived sources. EVs from these sources exhibit distinct biological activities, targeting properties, safety profiles, engineering potential, and degrees of clinical readiness, which influence their suitability for different therapeutic applications.

#### Stromal cells as a source of EVs

Among mammalian cells, mesenchymal stromal cells (MSCs) are widely regarded as a leading source for producing therapeutic EVs [99]. MSC-derived EVs (MSC-EVs) retain molecular features of both EVs and their parental MSCs. They commonly express EV-associated tetraspanins, including CD9, CD63, and CD81, along with MSC-related surface markers such as CD29, CD44, CD73, CD90, and CD105. Their biological effects are mainly attributed to the transfer of functional cargos into recipient cells. These cargos consist of growth factors, including FGF, HGF, and VEGF; anti-inflammatory and pro-angiogenic proteins; regulatory RNAs; and lipid mediators [144]. For instance, MSC-EVs have been shown to regulate macrophage responses under inflammatory conditions. In a recent study, human umbilical cord MSC-EVs (hucMSC-EVs) were taken up by lipopolysaccharide (LPS)-stimulated macrophages and subsequently shifted macrophage polarization from a pro-inflammatory M1 phenotype toward an anti-inflammatory M2-like state. Importantly, dexamethasone was successfully loaded into these EVs by electroporation, demonstrating their engineering flexibility as drug carriers for enhancing anti-inflammatory therapy [145].

Beyond their immunomodulatory effects, MSC-EVs play a vital role in tissue repair and regeneration. For instance, Wang et al. [146] isolated EVs from human adipose-derived MSCs (ADMSC-EVs) and showed that these EVs were readily absorbed by human keratinocytes. This uptake revitalized the keratinocytes and enhanced their ability to proliferate and migrate, and promoted the formation of new blood vessels (neovascularization) to support the healing tissues. Despite this enormous potential, the clinical translation of MSC-EVs faces a significant hurdle in the phenomenon of replicative senescence. As MSCs are expanded in culture to generate clinically relevant quantities, they can lose their therapeutic potency, and the produced EVs may become less effective [147]. To address such issues, researchers are exploring methods to rejuvenate senescent MSCs [148]. Induced pluripotent stem cells (iPSCs) are a promising alternative source, since they have unlimited capacity for self-renewal and can provide an endless, highly standardized source of therapeutic EVs. These iPSCs not only minimize the issue of donor variability and senescence but also bypass ethical concerns associated with other stem cell sources [149]. The therapeutic potential of iPSC-derived EVs (iPSC-EVs) has been reported by several studies. For instance, in a mouse model of ischemic stroke, human iPSC-EVs specifically targeted the damaged brain region and upregulated neuroprotective genes while downregulated inflammatory genes. When these EVs were loaded with brain-derived neurotrophic factor (BDNF), the therapeutic effect of these BDNF-loaded iPSC-EVs was even more pronounced, showing enhanced functional recovery and stronger neurogenesis [100].

Moreover, neural stem cells (NSCs) are the primary progenitors of the nervous system and produce EVs that are exceptionally adapted for the treatment of neurological conditions [150]. This demonstrates the principle of tissue-specific advantage, as neural stem cell-derived extracellular vesicles (NSC-EVs) are enriched with a cargo of specific miRNAs that actively support neurogenesis, offer neuroprotection, and help maintain the BBB. These properties make them a highly suitable therapeutic option for conditions like Alzheimer's and stroke [151]. NSC-EV therapeutic mechanisms are multifaceted. For example, they promote the expression of antioxidant enzymes and reduce reactive oxygen species (ROS) production, which helps to reduce mitochondrial dysfunction and prevent neuronal loss [152]. Despite its therapeutic potential, the primary limitation of NSC-EVs remains the significant ethical and logistical challenges associated with the human NSC source, which is a major barrier to their clinical translation.

#### Immune cell-derived EVs

Immune cells, including macrophages, DCs, T cells, B cells, and other lymphocytes, are also a major source of EVs [101]. Immune cell-derived EVs are the natural agents of immunosurveillance and play a central role in orchestrating the body's defense mechanisms [153]. Their function is highly context-dependent, as they can mediate both potent immune activation and targeted immune suppression, making them a key focus for therapeutic development [101]. Their cargo composition depends strongly on the parental cell type and functional state. These vesicles can carry cytokines, proteins, peptides, and regulatory nucleic acids [154]. For instance, neutrophil-derived MVs contain the anti-inflammatory protein annexin A1[155]. Natural killer cell-derived extracellular vesicles (NK-EVs) contain the cytotoxic protein perforin, granzyme B, as well as FasL, and carry TNF-related apoptosis-inducing ligand (TRAIL) [156]. Tumor-suppressive miRNAs are also present in NK-EVs [157]. Dendritic cell-derived extracellular vesicles (DC-EVs) can contain melanoma-associated antigen peptides [158]. Their most prominent applications are in cancer immunotherapy. As professional antigen-presenting cells, DC-EVs function as decentralized messengers of the immune system [159]. These EVs are loaded with peptide-MHC complexes, which can be transferred to other DCs and T-cells to amplify the antigen-presentation process and initiate a powerful and targeted antitumor response [160]. Moreover, in a glioma mouse model, neutrophil EVs loaded with doxorubicin (DOX) were able to cross the BBB and effectively target inflamed brain tumors, resulting in a significant inhibition of tumor growth [161]. However, despite their powerful mechanism, significant challenges remain for clinical translation, including the difficulty of sourcing and expanding primary immune cells on a large scale, as well as ensuring the safety of their potent cargo to prevent off-target autoimmune reactions.

#### Cancer cell-derived EVs

Cancer cells are one of the major sources of EVs, as they are immortal and secrete more EVs than normal cells. Cancer cell-derived EVs represent a double-edged sword in the context of therapeutic development. Despite their pro-tumor functions, their natural function is to promote cancer progression and metastasis by facilitating tumor growth, interfering with anticancer immunity, and establishing premetastatic niches in different organs. Nevertheless, these pro-cancer EVs possess a unique and powerful characteristic-natural tropism for their parental cancer cells. This inherent tropism can be harnessed, as recent research demonstrated that engineering cancer cell-derived EVs can transform into more potent anticancer agents [102].

One of the most valuable therapeutic applications of cancer cell-derived EVs is their engineering as personalized cancer vaccines. These cancer cell-derived EVs are naturally enriched with a diverse array of antigens from their parent tumor cells. They also serve as a rich source of tumor-associated antigens (TAAs) and tumor-specific antigens (TSAs) that are similar to their source cancer cells [162]. When cancer cell-derived EVs are taken up by antigen-presenting cells such as DCs, the tumor antigens are processed and presented on MHC molecules that, in turn, prime naïve T cells to recognize and attack the cancer cells [163]. However, native cancer cell-derived EVs often have limited immunogenicity as they contain pro-tumor cargoes. To address this issue, researchers have developed strategies to boost their immunostimulatory properties. For instance, applying heat stress to tumor/cancer cells before EV collection enriches the cancer cell-derived EVs with HSPs, including HSP70, which serves as a powerful natural adjuvant to boost the immune system and elicit a stronger antitumor immune response [164].

In addition to their application as cancer vaccines, the natural tropism of cancer cell-derived EVs makes them exceptional vehicles for targeted drug delivery. This property effectively turns them into Trojan horses that home to their cells of origin [165]. As cancer cell-derived EVs share similar membrane composition and surface proteins with their source cells, these cancer cell-derived EVs can be loaded with chemotherapeutic agents and selectively target both the primary tumor and distant metastatic sites [91]. Numerous chemotherapeutic agents like DOX, paclitaxel (PTX), and cisplatin have been successfully encapsulated into cancer cell-derived EVs and demonstrated excellent tumor-targeting efficiency in preclinical models [165, 166].

#### Blood cell-derived EVs

Blood is composed of two primary liquid portions: serum and plasma, both of which are sources of blood EVs [103]. The main difference between them is that the plasma is derived from anticoagulated blood, whereas serum is prepared from clotted blood. During the clotting process, a massive release of EVs from platelets takes place, which means that serum EVs do not accurately reflect the circulating state and differ significantly from plasma-derived EVs [167]. Blood is the primary source of liquid biopsies, and it makes it most crucial for the detection of rare EV populations that originate from different tissues and enter the circulating blood. This approach offers a real-time snapshot of patient health status. For instance, the presence of EVs derived from damaged cardiomyocytes in blood can serve as an indicator of cardiac injury [168].

Among circulating blood-cell EVs, red blood cell-derived EVs (RBC-EVs) are an abundant and therapeutically attractive population. Under normal physiological conditions, RBC-EVs account for approximately 7.3% of EVs in whole blood. Their production occurs naturally during erythrocyte maturation and aging, while vesiculation can also be stimulated by ATP depletion, calcium influx, oxidative stress, cytokines, complement activation, and high shear stress [169]. Their protein cargo includes hemoglobin and acetylcholinesterase [170]. As mature erythrocytes lack nuclei and mitochondria, RBC-EVs do not normally contain nuclear or mitochondrial DNA [171]. However, they contain substantial amounts of regulatory miRNAs [172]. RBC-EVs offer important manufacturing advantages because RBCs are abundant, readily obtained from donated blood, and can generate vesicles under control ex vivo or in vitro stimulation. RBCs from blood group O donors may provide a potential source for allogeneic RBC-EV production [170]. From a safety perspective, RBC-EVs exhibit high biocompatibility and low immunogenicity because of their erythrocyte origin [173]. Their lack of nuclear and mitochondrial DNA reduces the risk of unwanted horizontal gene transfer compared with EVs derived from nucleated cells [171]. CD47 expression may also extend their circulation by reducing macrophage-mediated clearance [174]. However, RBC-EVs generated during pathological conditions or prolonged blood storage may also possess pro-inflammatory, pro-coagulant, or vascular effects, making source selection and production control critical for therapeutic safety [175].

### Plant-derived EVs

One of the major sources for EVs are plants. Plants release nanovesicles known as plant-derived extracellular vesicles (PDEVs) that share structural and functional similarities with mammalian EVs [104]. Similar to human EVs, PDEVs are also divided into 3 major groups: exosomes range from 50 to 150 nm, whereas PM-derived MVs range from approximately 150 to 1000 nm [176] and ApoBDs 1000–5000 nm [128]. The membrane composition of PDEVs is distinct from that of mammalian EVs. Rather than being dominated by cholesterol-rich lipid-raft domains, PDEV membranes contain plant-specific lipids and cell wall-associated polysaccharides, including glycosylinositolphosphoceramides [110]. Their membranes are also enriched in phosphatidic acid, which can facilitate endosomal escape and cellular uptake [177]. Membrane-associated proteins such as PEN1 and the tetraspanin TET8 contribute to vesicle formation, structural organization, and molecular cargo incorporation [178].

PDEVs offer several distinct advantages over other EVs, which make them more attractive for therapeutic uses. As it is known, PDEVs are sourced from plants, and plants are sustainable, abundant, and a low-cost resource. PDEVs also bypass many of the issues related to mammalian EVs, such as ethical concerns and the extra cost of cell culturing [123]. Moreover, PDEVs are generally considered nontoxic and non-immunogenic to human health and are free from the risk of transmitting human pathogens [133]. Altogether, these properties enhance the safety profile of PDEVs. In addition, PDEVs have a unique cargo composition that includes lipids, proteins, nucleic acids, defense-related small RNAs, antimicrobial proteins, hydrolytic enzymes, antioxidant enzymes, and plant-specific metabolites. The cargo composition varies depending on the plant source, and this source-dependent cargo contributes substantially to their biological and therapeutic activities. For example, ginger-derived EVs contain bioactive components such as 6-gingerol [179] and the antioxidant enzyme copper–zinc superoxide dismutase [180], whereas turmeric-derived EVs carry curcumin [181]. PDEVs also transport plant miRNAs, including osa-miR164d from ginger-derived EVs [116], miR-5781 from soybean-derived EVs [117], and Rgl-exomiR-7972 from Rehmannia glutinosa root-derived EVs [118]. This unique composition has been linked to a wild array of biological functions such as the anti-inflammatory effect of turmeric PDEVs in colitis model by modulating pro-inflammatory cytokines and by inactivating the nuclear factor kappa-B (NF-κB) pathway [181]. In addition, the anticancer potential of PDEVs is another major research area. For instance, EVs derived from bitter melon have been shown to suppress breast cancer cell proliferation and migration and promote ROS production. This increase in ROS triggers oxidative stress-mediated cell death [182].

Some edible and medicinal PDEVs have been reported to show preferential uptake by intestinal cells, gut microbiota, immune cells, or inflamed tissues. For example, turmeric-derived EVs showed targeted therapeutic effects in ulcerative colitis models [183], while ginger-derived EVs were reported to accumulate in inflamed intestinal mucosa and promote mucosal healing in inflammatory bowel disease and colitis-associated cancer models [179]. In addition, lipid composition may contribute to selective interactions with gut microbiota, as PDEV-associated miRNAs and lipid components from ginger-derived EVs have been shown to shape gut microbiota composition [119]. However, this natural targeting capacity should be interpreted carefully because most evidence remains preclinical and source-dependent. The possible molecular basis may involve plant-specific lipid composition, surface-associated proteins, plant miRNAs, and secondary metabolites. If further validated, this property may strengthen the use of PDEVs as oral and gut-targeted delivery platforms compared with some mammalian EVs.

Moreover, PDEVs also show considerable engineering flexibility. Exogenous therapeutic cargo can be incorporated through different loading methods. For instance, electroporation is suitable for loading nucleic acids, whereas sonication can achieve high loading efficiency for lipophilic compounds [128]. Their surfaces can additionally be functionalized with targeting ligands; for example, folic acid-modified ginger-derived EVs were developed to enhance recognition of folate receptor-expressing macrophages [184]. Apart from therapeutic effects, PDEVs also play a major role as nanocarriers for drug delivery. PDEVs have exceptional stability in the acidic and enzymatic environment of the gastrointestinal tract (GIT), which makes them ideal candidates for oral drug delivery of different biomolecules. A recent clinical trial also demonstrated enhanced oral delivery and bioavailability of curcumin by PDEVs in colon cancer treatment (ClinicalTrials.gov Identifier: NCT01294072).

### Bacteria-derived EVs

Bacteria are one of the emerging sources of therapeutic EVs. Bacteria-EVs are generally nanosized, spherical vesicles enclosed by lipid-bilayer membranes released by both Gram-positive and Gram-negative bacteria. They serve as the primary mode of communication between host and bacteria. Bacteria as a source of EVs offer several advantages. First, bacteria grow faster than mammalian cells and are more cost-effective. Their cultivation on an industrial scale is much easier due to optimized protocols, straightforward genetic modification, and simple EV isolation techniques [105].

EVs isolated from Gram-positive bacteria have been reported to range from approximately 20 to 100 nm [107]. However, bacteria-EV size can vary according to parental strain, growth stage, biogenesis pathway, and culture conditions. Vesicles measuring 20–100 nm are preferentially internalized through caveolae-mediated endocytosis, whereas vesicles larger than 100 nm (approximately 90–450 nm) may enter recipient cells through macropinocytosis or other endocytic pathways [108]. The structure of bacteria-EVs depends on the cell-envelope organization of the parental bacterium. Gram-negative bacteria generate outer membrane vesicles (OMVs) through outward budding of the outer membrane [185]. In contrast, Gram-positive bacteria release cytoplasmic membrane vesicles when the cytoplasmic membrane protrudes through pores formed in the thick peptidoglycan layer [186]. The membrane of bacteria-EVs contains several bacterial phospholipids and membrane-associated components such as phosphatidylethanolamine, phosphatidylinositol [111], phosphatidylglycerol, and cardiolipin [112]. OMVs additionally contain LPS, including lipid A, core oligosaccharides, and O-antigens [113]. Their membranes may also contain outer membrane proteins such as OmpA, OmpC, OmpF, OmpT, OmpU, and TolC [105]. Gram-positive bacteria-EVs may contain cytoplasmic membrane components together with peptidoglycan-associated molecules.

The molecular cargo of bacteria-EVs includes proteins, enzymes, lipids, carbohydrates, metabolites, DNA, and RNA. Their protein cargo contains periplasmic proteins and enzymes [120]. InsP6 phosphatase has been identified as a metabolism-regulating enzyme within bacterial EVs [187]. *β*-Lactamase can also be transported by bacterial EVs and contribute to antibiotic resistance [121]. Some pathogenic bacteria-EVs carry Shiga toxin [122]. Hemolytic phospholipase C has also been detected in bacterial vesicles [188]. Other virulence-associated components include hemolysins, leukocidins, proteases, and exfoliative toxins [189]. In addition, bacteria-EVs can transport bacterial DNA and RNA to recipient cells or other bacteria. Bacteria-EVs are also reported with diverse therapeutic effects. For instance, probiotic bacteria-EVs sourced from *Bacteroides fragilis* can induce interleukin 10 (IL-10) secretion from immune cells via a toll-like receptor 2 (TLR2)-dependent pathway and can promote an anti-inflammatory environment [188, 190]. EVs from pathogenic bacteria like *Pseudomonas aeruginosa* can trigger a strong pro-inflammatory response, underscoring their dual role in immunity [126]. Beyond immunomodulation, another exciting area in which bacterial EVs play a role is neurology. Certain bacteria-EVs possess the natural ability to cross the BBB. This property enables their use as trojan horse delivery vehicles to carry neuroprotective drugs directly into the brain to treat conditions like ischemic stroke [191]. Moreover, GSK's Bexsero®, an EV-based vaccine derived from bacteria, is approved and has already demonstrated a safety profile and efficacy of bacterial EVs in humans [131].

From a production perspective, bacteria offer rapid proliferation, mature fermentation systems, diverse gene-editing tools, and relatively high EV production yields. However, EV yield and composition vary with the bacterial strain, culture conditions, growth phase, and genetic background. For example, early-stage Bacteroides thetaiotaomicron EVs are mainly produced non-lytically and contain more lipoproteins, whereas late-stage vesicles are primarily generated through lysis and may contain cargo that is less selectively packaged [134]. Careful control of culture conditions is therefore essential for consistent production. Moreover, bacteria-EVs are highly engineerable. Parental bacteria can be modified to reduce toxicity, increase vesicle production, or display targeting and therapeutic proteins. CRISPR-Cas9 can decrease endogenous protein content and increase cargo-loading space [129], while recombinant DNA technology enables the display of antigens or functional proteins on EV surface [124]. Isolated EVs can also be loaded by electroporation, sonication, freeze–thaw treatment, or incubation. Surface modification can further improve tissue targeting [105]. However, most engineered bacteria-EV systems for drug delivery and regenerative medicine remain preclinical and require standardized manufacturing, endotoxin control, potency testing, biodistribution studies, and long-term safety evaluation.

## Advantages of EVs as next-gen therapeutic delivery platforms

The growing interest in EVs as therapeutic delivery systems is largely driven by their ability to combine the advantages of natural biological carriers with the flexibility of engineered nanomedicine [192, 193]. Conventional delivery systems, including liposomes, lipid nanoparticles (LNPs), polymeric nanoparticles, inorganic nanoparticles, and viral vectors, have contributed significantly to modern drug and gene delivery. However, these platforms still face important limitations related to toxicity, immune recognition, inefficient tissue targeting, premature cargo release, limited biological-barrier penetration, and safety concerns during repeated administration [192, 194, 195]. In this context, EVs offer a biologically derived alternative that can transport diverse therapeutic cargoes while retaining many features of endogenous intercellular communication.

One major advantage of EVs is their intrinsic biocompatibility and low immunogenicity [196]. Since EVs are naturally released by cells and are composed of endogenous lipids, proteins, glycans, and nucleic acids [197], they are generally better tolerated than many synthetic or inorganic nanocarriers. Liposomes and LNPs can be engineered for improved circulation and cargo delivery, but their lipid composition, surface charge, and repeated dosing can trigger immune recognition, complement activation, or accelerated clearance [194, 195, 198, 199]. Inorganic nanoparticles, such as metallic, silica, or carbon-based nanoparticles, may provide high stability and controlled-release properties, but their limited biodegradability and potential long-term tissue accumulation raise additional safety concerns [192]. In contrast, EVs are biodegradable [197], and can be derived from selected cellular sources, including autologous or less immunogenic allogeneic cells, to improve safety and reduce immune-related complications [200, 201].

EVs also possess natural targeting and uptake properties that distinguish them from many synthetic delivery platforms. Conventional nanoparticles often depend on passive accumulation, such as the enhanced permeability and retention effect [202], or require additional surface modification with ligands, antibodies, peptides, or polymers to improve targeting [203, 204]. Although these strategies can enhance delivery, they may also increase production complexity, cost, and potential safety risks [204, 205]. EVs naturally carry membrane proteins, integrins, tetraspanins, adhesion molecules, and glycans that support interactions with specific recipient cells and tissues. These native surface features can contribute to tissue tropism, immune evasion, cellular uptake, and navigation of biological barriers. Moreover, the surfaces of EVs can be further engineered to display targeting ligands or immune-modulatory signals without completely disrupting their natural membrane architecture [206, 207].

Another important feature of EVs is their capacity to protect and deliver a wide range of therapeutic cargoes. Their lipid bilayer shields sensitive molecules, including RNAs, proteins, peptides, cytokines, and small-molecule drugs, from enzymatic degradation in extracellular environments [18]. Compared with some lipid-based or polymeric nanoparticles, which may suffer from instability, cargo leakage, or premature cargo release [192], EVs provide a stable biological compartment that can preserve cargo bioactivity during circulation [193]. EVs can also deliver cargo through multiple routes, including receptor-mediated binding, endocytosis, membrane fusion, and local cargo release near recipient cells [208]. These mechanisms make EVs particularly attractive for delivering nucleic acids, proteins, and small molecules in the treatment of diseases involving difficult-to-reach tissues.

Compared with viral vectors such as adenoviral systems, EVs may also offer safety advantages for repeated or cell-free therapeutic delivery [209]. Viral vectors are highly efficient for gene transfer but may be limited by immunogenicity, pre-existing antiviral immunity, packaging constraints, inflammatory responses, and concerns related to repeated administration [210, 211]. EVs do not contain viral replication machinery and can deliver RNAs, proteins, and gene-modulatory molecules with potentially lower immunogenicity [212]. Although their gene-transfer efficiency may be lower than that of some viral vectors [193], their safety profile, cargo versatility, and engineering adaptability make them attractive for applications where repeated dosing or transient modulation is preferred.

Overall, EV-based delivery systems show natural biocompatibility, intrinsic targeting capacity, cargo protection, engineering flexibility, and therapeutic functionality. Therefore, EVs should not be considered universally superior to synthetic or viral delivery platforms, but rather as biologically inspired carriers with distinct advantages for delivering therapeutic diverse cargoes.

## Isolation and purification strategies for EVs

**Fig. 2:**
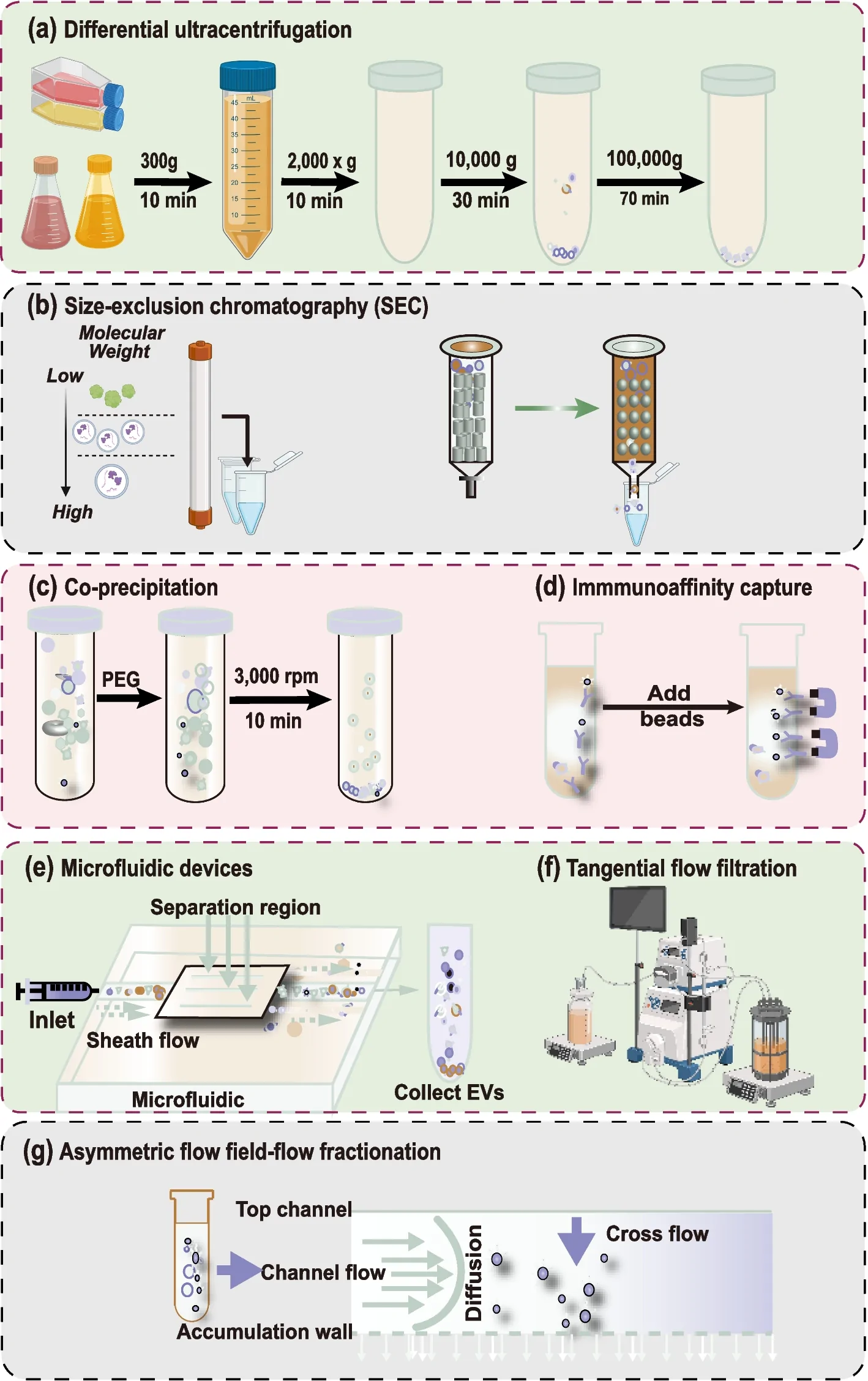
Common techniques used for the isolation and purification of EVs**. a** Differential ultracentrifugation: Stepwise centrifugation at increasing speeds (300 × g, 2,000 × g, 10,000 × g, and 100,000 × g) separates cells, debris, microvesicles, and exosomes based on size and density. **b** Size-exclusion chromatography (SEC): EVs are separated according to molecular size as they pass through a porous resin column, allowing larger vesicles to elute earlier while smaller molecules are retained. **c** Co-precipitation: PEG (Polyethylene glycol) is added to aggregate EVs, followed by low-speed centrifugation (3,000 rpm, 10 min) to pellet the vesicles. **d** Immunoaffinity capture: EVs expressing specific surface markers are selectively isolated using antibody-coated magnetic beads. **e** Microfluidic devices: EVs are isolated using microchannels that leverage controlled flow dynamics (inlet/sheath flow) to separate vesicles based on size or physical properties. **f** Tangential flow filtration: A membrane-based method in which flow runs parallel to the filter surface, enabling concentration and purification of EVs while removing small contaminants. **g** Asymmetric flow field-flow fractionation: EVs are separated in a channel using cross-flow forces, with smaller particles eluting earlier and larger vesicles retained near the accumulation wall

The efficient isolation and enrichment of EVs from their source represents a critical and foundational step for EV applications in biomedical research [213]. The selected method for isolation directly influences EVs recovery, purity, structural integrity, cargo preservation, and the reliability of downstream molecular and functional analyses. This is especially important for therapeutic EV development, where reproducibility, scalability, safety, and batch-to-batch consistency are essential. Conventional methods used for EV isolation include differential ultracentrifugation and density-gradient ultracentrifugation for density-based separation. Ultrafiltration (UF), size exclusion chromatography (SEC), and tangential-flow filtration (TFF) for size-based fractionation. Immunoaffinity capture for antigen-specific enrichment, and polymer-based precipitation for solubility-based concentration. In addition, advanced approaches such as microfluidics, asymmetric flow field-flow fractionation (AF4), and lipid membrane-based capture have been developed to improve workflow speed, purity, specificity, and translational applicability (Fig. 2) [214]. A comparative overview of these isolation and purification strategies, including their principles, recovery, purity, scalability, major advantages, and limitations is provided in Table 2.

**Table 2:** Comparative overview of isolation and purification strategies for therapeutic EV development

Method	Principle	Recovery	Purity	Effect on EV integrity	Scalability	Main advantages	Main limitations	Therapeutic relevance	Reference
Differential ultracentrifugation	Sequential centrifugation separates EVs according to size, density, and sedimentation rate	Moderate	Low to moderate	High centrifugal force may affect vesicle morphology and biological activity	Moderate	Widely used, reagent-free, inexpensive, and suitable for initial EV enrichment from large sample volumes	Time-consuming and often co-isolates protein aggregates, lipoproteins, and nucleic acid–protein complexes	Useful as an initial enrichment step, but usually requires further purification for therapeutic-grade EVs	[215]
Density-gradient ultracentrifugation	EVs migrate to specific density fractions in sucrose or iodixanol gradients	Low to moderate	High	Better preservation than repeated crude ultracentrifugation, but prolonged processing may still affect EV quality	Low	Produces cleaner EV fractions and separates vesicles from contaminants with different buoyant densities	Labor-intensive, technically demanding, time-consuming, and difficult to scale	Suitable for high-purity EV preparation, especially for proteomic and functional studies	[214, 216–219]
Size-exclusion chromatography	EVs are separated from smaller soluble proteins and free nucleic acids using a porous gel matrix	Moderate	High	Gentle method that preserves EV structure and biological activity	Moderate	Provides high-purity EVs with minimal protein contamination and good functional preservation	EV fractions are often diluted and may require a concentration step	Useful as a polishing method after initial enrichment or concentration	[220, 221]
Ultrafiltration	Semi-permeable membranes retain EVs while smaller soluble molecules pass through	Moderate to high	Low to moderate	Shear stress and membrane pressure may damage vesicles or promote aggregation	Moderate	Simple, rapid, and useful for concentrating diluted EV-containing samples	Membrane clogging, reduced recovery, aggregate formation, and possible vesicle deformation	Suitable for sample concentration before further purification	[222–224]
Tangential-flow filtration	Sample flows parallel to the membrane surface, reducing clogging during size-based concentration	High	Moderate	Generally gentle and better preserves EV integrity than conventional dead-end filtration	High	Scalable, reproducible, and suitable for processing large volumes	Requires specialized equipment and process optimization	Highly suitable for therapeutic EV manufacturing, especially for concentration and buffer exchange	[104, 225]
Polymer-based precipitation	Hydrophilic polymers such as PEG reduce EV solubility and precipitate EV-rich fractions	High	Low	Generally mild, but polymer residues and contaminants may affect downstream function	High	Simple, low-cost, scalable, and compatible with large-volume samples	Low specificity and frequent co-precipitation of proteins, polymers, and other non-EV particles	Useful for rapid enrichment when yield is prioritized, but less suitable alone for clinical-grade EVs	[222, 226, 227]
Immunoaffinity capture	Antibodies or aptamers bind specific EV surface markers to isolate selected EV subpopulations	Low to moderate	Very high	Usually preserves captured EVs, although elution conditions may affect vesicle quality	Low	Highly specific and useful for isolating tissue-, disease-, or lineage-specific EVs	Expensive, low throughput, marker-dependent, and may miss EVs lacking the selected antigen	Valuable for biomarker discovery, liquid biopsy, and quality control of defined EV subpopulations	[228–230]
Microfluidic isolation	EVs are isolated in microchannels using immunoaffinity, size filtration, dielectrophoresis, viscoelastic flow, or nanotechnology-assisted capture	Moderate	Moderate to high	Usually gentle because only small sample volumes are processed	Low to moderate	Rapid, sensitive, automated, and compatible with small clinical samples	Device fabrication, operational complexity, and limited large-scale validation remain challenges	Promising for clinical diagnostics and rapid EV analysis from limited sample volumes	[231–239]
Asymmetric flow field-flow fractionation	Label-free hydrodynamic separation uses channel flow and cross-flow to resolve EV subpopulations by size	Moderate	High	Preserves EVs in a near-native state because no antibody or polymer precipitation is required	Low	High-resolution separation and useful for analyzing EV heterogeneity	Requires specialized instrumentation and technical expertise	Best suited for analytical fractionation and characterization of EV subtypes	[240–242]
Lipid membrane-based capture	EVs are captured through common lipid-bilayer features, such as phospholipid phosphate groups or lipid-labeling chemistry	High	Moderate to high	Potentially gentle, but effects on EV function require further validation	Moderate	Rapid, high-recovery, and does not depend on cell-specific protein markers	Still emerging and requires broader validation across sample types	Promising for rapid EV capture from complex samples, but not yet fully standardized for therapeutic production	[222, 243–245]

EVs isolation often follows a two-step design. In mammalian EVs studies, a heterogeneous EVs solution is purified by methods such as ultracentrifugation or SEC that help to remove cells, debris, and other non-EVs components [213], followed by more selective approaches such as immunoaffinity capture, when specific EV subpopulations are needed. This strategy is particularly useful for isolating tissue- or lineage-specific EVs, such as neuronal or glial EVs from blood, using antibodies against defined surface markers [246]. In plant EVs research, the initial materials for EVs may include tissue homogenates, juices, or apoplastic wash fluids. Tissue homogenates usually provide higher EV yields but introduce intracellular contamination, whereas apoplastic wash fluids offer cleaner preparations but lower EV concentrations. After the initial collection of EV-containing fluids, ultracentrifugation is the principal enrichment step, which can be followed by SEC or TFF to obtain analytical-grade purity [213].

### Ultracentrifugation-based methods

Ultracentrifugation-based methods remain among the most widely used approaches for initial EV enrichment. This method is designated as the "gold standard" in the field mainly because of its long-standing use, easy accessibility, and capacity to process large sample volumes without specialized reagents [247]. The fundamental principle of the ultracentrifugation technique is the separation of EV particles from a solution based on their size, density, and sedimentation rate when subjected to high centrifugal forces [248]. Differential ultracentrifugation usually provides crude EV enrichment rather than highly purified EV preparations. Its major limitation is the co-sedimentation of non-EV contaminants, including protein aggregates, lipoproteins, and nucleic acid–protein complexes [215]. Density gradient ultracentrifugation, a more refined ultracentrifugation technique, is used to separate particles based on their buoyant density and offers significantly higher purity [216]. In this method, a pre-enriched EV sample, is layered onto a density gradient medium normally made up of sucrose or iodixanol [217], and then subjected to a high-speed ultracentrifugation, allowing EVs to migrate to density-corresponding fractions [218]. Compared with differential ultracentrifugation, this method improves EV purity and is particularly useful for applications that require cleaner preparations, such as proteomic analysis [219]. However, density-gradient ultracentrifugation is labor-intensive, technically demanding, and time-consuming [214].

### Size-based methods

Size-based isolation methods —including size-exclusion chromatography (SEC), ultrafiltration (UF), and tangential flow filtration (TFF)— separate or enrich EVs based on particle size or hydrodynamic radius. These methods are widely used because they can preserve vesicle integrity and support EV concentration or purification from large-volume samples.

SEC operates on a porous gel matrix, where larger vesicles elute earlier, while smaller contaminants-such as soluble proteins and free nucleic acids-elute later, producing EV preparations with well-preserved integrity and biological activity [220]. However, SEC tends to dilute EVs across multiple fractions; therefore, it is commonly employed as a polishing step after initial enrichment to improve purity [221].

UF uses semi-permeable membranes with defined molecular weight cut-offs (MWCO) to retain EVs while allowing smaller soluble molecules to pass through, which is particularly effective for concentrating diluted samples [222, 223]. However, conventional dead-end UF may result in membrane clogging, reduced recovery, aggregate formation, and possible vesicle damage caused by shear stress [224].

TFF overcomes some of these limitations by directing the sample flow parallel to the membrane surface, reducing clogging and enabling more uniform processing [225]. Compared with traditional UF and ultracentrifugation, TFF is more scalable, gentle, and reproducible, making it particularly attractive for therapeutic EV production [104].

### Polymer-based precipitation

Polymer-based precipitation is widely adopted to isolate EVs by altering their solubility within a sample [222], and is mostly applied in isolating EVs from plant sources [226]. The core principle of the technique involves the addition of a hydrophilic polymer, mostly polyethylene glycol (PEG), which creates a dense network in the solution and precipitates EVs out of the solution. The obtained EV-rich precipitate can then be collected using low-speed centrifugation [227]. The simple protocol of the technique, along with low cost and high scalability, makes it a popular choice. This method is commonly used for large-volume samples and forms the basis of numerous commercial EV isolation kits [222].

### Advanced approaches

Immunoaffinity capture is a highly specific isolation method that enables the selective extraction of distinct EV subpopulations from complex samples [228]. Unlike density- or size-based methods, this approach utilizes the specific biochemical interaction between antibodies and antigens present on the surface of EVs. The core principle of this method is based on immobilizing antibodies onto a solid support, which then binds to EVs expressing the corresponding surface antigen [229]. This technique is not only limited to using antibodies; other bio-affinity agents like aptamers can also be used for capturing EVs [230]. The major advantage of immunoaffinity capture is its ability to isolate specific EV subpopulations with high purity, making it valuable for biomarker discovery, liquid biopsy, and quality control of therapeutically relevant EV preparations.

Microfluidic technology has emerged as a powerful and innovative approach for EV isolation and offers significant advantages for clinical applications [231]. These "lab-on-a-chip" systems manipulate fluids within precisely engineered microchannels that enable rapid and highly efficient purification of EVs from samples with small volumes such as tears, saliva, or cerebrospinal fluid [232]. Microfluidic EV isolation can be achieved through several principles, including immunoaffinity capture [233, 234], size-based filtration [235, 236], dielectrophoresis [237], viscoelastic separation [238], and nanotechnology-assisted capture [239]. By integrating separation, enrichment, and detection steps into a single platform, microfluidic technology can reduce processing time, enhance sensitivity, and improve reproducibility compared to traditional methods. The technique also plays a pivotal role in advancing our understanding of EV biology and realizing their full potential in clinical diagnostics and therapeutics.

AF4 is a hydrodynamic, label-free separation method that enables size-based sorting and high-resolution analysis of EV populations within complex biological samples, allowing direct visualization of heterogeneity within these populations [240]. The basic principle of AF4 involves the interaction between two mobile phases: the horizontal flow through the channel, and the vertical cross-flow. This dual-phase system allows for efficient separation of nanoscale particles [241]. A major advantage of AF4 is its ability to separate EV subpopulations that differ by small sizes while preserving their native state, since it does not require antibody binding or polymer-based precipitation [242].

Lipid membrane-based isolation is an emerging, innovative approach that takes advantage of the unique biochemical properties of the EV lipid bilayer. The EV lipid bilayer consists of amphiphilic phospholipids with hydrophilic phosphate heads and hydrophobic tails [243]. This characteristic provides a distinct opportunity for EV capture and isolation through specific chemical interactions. One representative strategy uses metal oxides, such as titanium dioxide (TiO₂), which can bind phosphate groups on EV membrane phospholipids through chemisorption [222]. This chemisorption mechanism has been successfully applied to rapidly isolate EVs from complex human samples [244]. Another approach combines lipid labeling and click chemistry [245]. The main advantages of lipid membrane-based isolation are rapid processing, high recovery, and the ability to target a fundamental structural feature shared by EVs. However, these methods are still relatively new and require further optimization, validation across diverse sample types, and assessment of their effects on EV integrity and downstream functional assays.

Overall, for the development of therapeutic EVs, isolation strategies should be selected based on the EV source, intended application, and required balance between recovery, purity, scalability, and vesicle integrity. Since no single method can fully satisfy all requirements for clinical-grade EV preparation, combined workflows are often preferred. For example, UF or TFF can be used for initial concentration, followed by SEC or density-gradient ultracentrifugation to improve purity, whereas immunoaffinity capture can be used when specific EV subpopulations are required. Source-specific and standardized isolation workflows are therefore essential for improving the reproducibility, safety, and translational reliability of EV-based therapeutics.

## Characterization strategies for EVs

**Fig. 3:**
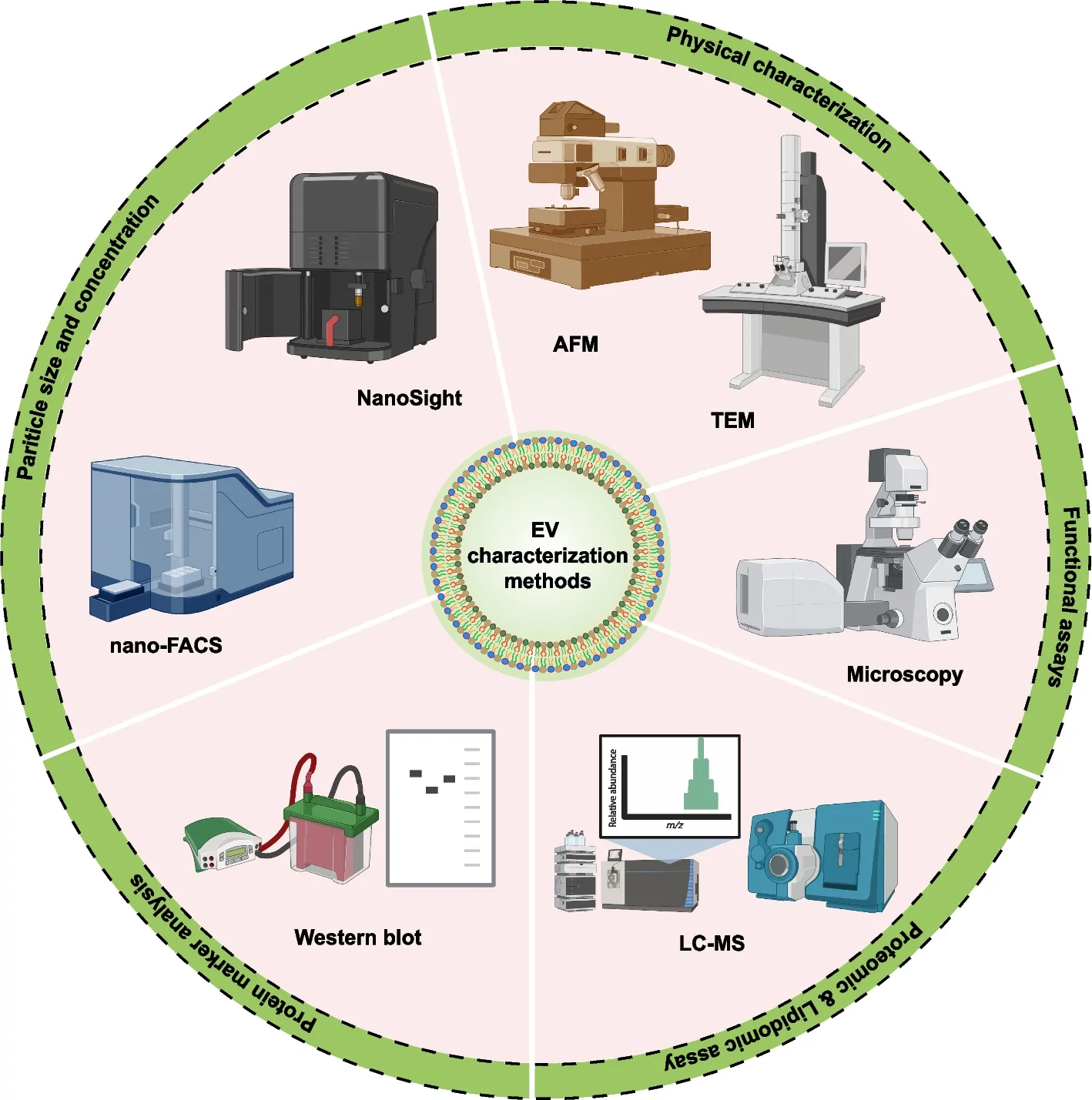
Techniques used for the physical, biochemical, and functional characterization of EVs. EV particle size and concentration can be determined using NanoSight and nano-FACS. EV morphology and surface topography can be examined by TEM and AFM, respectively. Western blotting is used to detect EV-associated protein markers, whereas LC–MS enables proteomic and lipidomic profiling. Microscopy-based assays can further assess EV localization, cellular uptake, and interactions with recipient cells

After successful isolation of EVs from biological samples, comprehensive characterization is crucial to confirm their presence, purity, and specific properties. Due to the complex nature of EVs, no single method is sufficient for complete analysis; therefore, a combination of different techniques is necessary. This validation is essential for the reproducibility and reliability of downstream applications. The characterization techniques are broadly classified into physical and chemical characterization, which are discussed below (Fig. 3).

### Physical characterization

Physical characterizations of EVs primarily focuses on their morphology, size distribution, and concentration. Techniques such as electron microscopy are used to examine EV morphology, whereas nanoparticle tracking analysis (NTA), dynamic light scattering (DLS), and tunable resistive pulse sensing (TRPS) are employed to determine their size distribution and concentration.

#### Microscopy

Microscopy plays a central role in understanding the morphology and structural organization of EVs. Several high-resolution imaging tools, such as transmission electron microscopy (TEM), scanning electron microscopy (SEM), cryo-electron microscopy (Cryo-EM), and atomic force microscopy (AFM), are widely used to characterize EVs' shape, size, surface topology, and internal structure. Each of these techniques operates on distinct physical principles and offers distinct advantages, as well as providing valuable insights into the physical nature of EVs.

TEM is one of the most widely used imaging methods for EV characterization because it enables high-resolution visualization of individual vesicles and helps distinguish EVs from non-vesicular contaminants [249, 250]. When TEM is combined with negative staining techniques, it characteristically reveals a "cup-shaped" morphology of the EV, which is an artifact of the sample preparation process [251]. Furthermore, its specificity can be enhanced through immunogold labeling, a method that uses gold-conjugated antibodies to accurately locate specific protein markers on the surface of EVs [249, 252]. In contrast to TEM, SEM provides detailed surface topography and spatial distribution of biological nanostructures [253]. SEM offers realistic 3D views that help to assess vesicle aggregation and surface patterning. However, both TEM and SEM require sample preparation steps such as fixation, dehydration, or coating, which may alter vesicle morphology [254]. Another technique, Cryo-EM, represents a significant advancement in imaging and operates on the principle of cryogenic preservation. This method involves rapid freezing of samples, which maintains the EVs in their natural-hydrated state and circumvents the need for chemical fixation or staining procedures that can introduce artifacts [255, 256]. In EV research, Cryo-EM allows a much more accurate visualization of vesicle morphology, membrane structure, and lipid bilayer organization [257, 258].

AFM is a powerful scanning probe technique that operates by physically scanning a surface with a nano-scale probe. This mechanism allows for the non-destructive characterization of the structure and mechanics of biological samples, often in their native aqueous environment, with high spatiotemporal resolution. In EV research, AFM is used to generate 3D morphological maps showing a spherical shape with diameters ranging from 30 to 50 nm [259]. A unique capability of AFM is its application in force spectroscopy [260], which can acquire data on the mechanical properties of EVs, such as their stiffness. Another microscopic method, super-resolution microscopy (SRM), encompasses a set of advanced imaging techniques designed to overcome the diffraction limit of light, which traditionally restricts the resolution of optical microscopy. In EV research, SRM provides the ability to visualize nanometric details of vesicle morphology, size, molecular composition, and the distribution of surface markers, which is crucial for the precise discrimination of EV subtypes [261].

Overall, microscopy is valuable for assessing EV morphology and integrity, but it should be combined with quantitative and molecular assays to ensure reliable characterization of therapeutic EV preparations.

#### DLS and NTA

DLS [262] and NTA [263] are widely used optical methods for evaluating EV size distribution and sample heterogeneity. DLS technique is based on the interaction between light and particles undergoing Brownian motion in a liquid medium. It is relatively simple and rapid, and requires no chemical labeling or extensive sample preparation. In the context of EV research, DLS has become a routine method for evaluating size distribution, dispersion uniformity, and colloidal stability of vesicle samples. Moreover, DLS can measure surface charge by zeta potential [264], which can help predict the stability and tendency of the suspension to aggregate. NTA, in contrast, enables the detection and tracking of individual particles, allowing precise measurement of both particle size and concentration [265], making it a widely adopted technique for EV characterization. However, its accuracy is highly dependent on sample purity, necessitating careful sample preparation.

#### TRPS

TRPS is a nanopore-based analytical technique specifically designed for single-particle characterization. It provides high-precision measurement of EV concentration, size, and surface charge [266]. The method is based on transient changes in electrical resistance when individual particles pass through a nanopore in an electrolyte solution [267]. As each EV crosses the pore, it generates an electrical pulse, allowing simultaneous estimation of particle size and concentration [268]. In EV research, TRPS has emerged as a powerful platform for both quantitative and multiparametric analysis, particularly in biomedical research and clinical translation, where precise size and concentration measurements are critical [269]. Furthermore, TRPS can assess the surface charge characteristics by calculating zeta potential [270]. Its simple operation, minimal sample requirement, and fast detection make it highly suitable for high-throughput and reproducible analysis [271]. However, TRPS has certain limitations. Its detection range may restrict sensitivity for very small EVs, and measurements can be affected by ionic current fluctuations, improper nanopore calibration, sample aggregation, and inadequate sample handling [272, 273].

### Molecular and biochemical characterization

Molecular and biochemical characterization is essential for confirming EV identity, cellular origin, surface markers, cargo composition, and sample purity. These analyses complement physical characterization by determining whether isolated particles carry expected EV-associated proteins, nucleic acids, lipids, and other bioactive molecules. Protein-based methods such as western blotting (WB) and enzyme-linked immunosorbent assay (ELISA) are commonly used to detect EV-associated markers and evaluate potential contaminants. Additionally, flow cytometry allows for the high-throughput analysis of surface proteins on individual EVs, which helps identify a specific EV subpopulation. Polymerase chain reaction (PCR)-based methods are commonly used to analyze nucleic acid cargo, whereas mass spectrometry (MS) enables comprehensive protein profiling. Moreover, advanced detection methods such as surface plasmon resonance (SPR), surface-enhanced Raman scattering (SERS), and total internal reflection fluorescence (TIRF) microscopy can improve the sensitivity and specificity of EV analysis.

#### Immunoassay-based protein analysis

Protein-based analytical techniques remain fundamental for confirming EV molecular identity, cellular origin, and purity [274]. WB and ELISA are the most frequently employed immunoassay-based techniques. Both rely on highly specific antigen–antibody interactions to detect target proteins with precision and sensitivity [275]. In EV research, WB serves as a standard validation step to confirm EV-specific markers and evaluate sample purity [276]. According to the Minimal Information for Studies of Extracellular Vesicles 2023 (MISEV2023) guidelines, EV characterization should include representative markers from transmembrane proteins, cytoplasmic proteins, and non-vesicular co-isolated structures (NVEPs), allowing both EV-associated proteins and potential contaminants to be evaluated [41]. This provides a molecular fingerprint that distinguishes EVs from other extracellular particles. Unlike WB, ELISA relies on specific antigen–antibody interactions. It enables quantitative detection of selected EV proteins and is valuable for biomarker screening, functional studies, and high-throughput analysis [241]. Advanced formats, such as droplet digital ELISA, can further improve detection sensitivity and specificity [277].

#### Quantitative polymerase chain reaction

Quantitative polymerase chain reaction (qPCR) is a fluorescence-based nucleic acid amplification technique that enables the detection and quantification of low-abundance genetic material with remarkable precision and speed. By continuously monitoring fluorescence signal dynamics during amplification cycles, this technique allows real-time measurement of nucleic acid concentration and completes the analysis within a few hours [278]. Due to its high sensitivity, specificity, and reproducibility, qPCR has become a fundamental tool for investigating EV-associated nucleic acids and elucidating their role in various physiological and pathological processes [279, 280]. Since EVs often contain very small amounts of RNA and DNA, several signal amplification strategies have been integrated into qPCR workflows to improve detection sensitivity. These include strand displacement reaction (SDR), hybridization chain reaction (HCR), rolling circle amplification (RCA), Clustered Regularly Interspaced Short Palindromic Repeats/CRISPR-associated proteins (CRISPR/Cas)-based amplification, and catalytic hairpin assembly, all of which amplify weak nucleic acid signals and enable reliable quantification from limited EV samples [208]. However, a persistent challenge in EV research is the lack of universally applicable and stably expressed reference genes (RGs), as inconsistent reference gene selection can compromise data comparability and reproducibility across studies. To address this issue, recent studies have identified several stably expressed RGs (*e.g*., SNRPG, OST4) that show stable expression across different EV types [281]. The establishment of such universal EV RGs is expected to promote the broader use of qPCR for the analysis of EV-related RNA cargo.

#### Flow cytometry

Flow cytometry is widely used for EV phenotyping due to its single-particle analysis and multi-parameter detection. However, conventional flow cytometry faces significant challenges with the small size and the heterogeneous nature of EVs. The method is often constrained by the lower limit of detection (LoD), which can bias size distribution analysis. Thus, establishing the LoD for each instrument is critical for reliable data interpretation [282, 283]. Advances—including enhanced side-scatter sensitivity, improved fluorescent labeling, and the development of nano-flow cytometry (nFCM) systems (*e.g.*, CytoFLEX and Apogee) –have significantly improved the detection of EVs in the 100–200 nm range [284]. nFCM allows simultaneous measurement of physical properties and surface markers at the single vesicle level and provides direct evidence of EV heterogeneity. It also demonstrating that lipophilic dye labeling does not interfere with surface marker detection, thereby supporting optimized staining protocols [285]. Thus, flow cytometry is valuable for EV subpopulation analysis, marker validation, and quality control of therapeutic EV preparations.

#### Mass spectrometry

MS is a cornerstone technique for the comprehensive molecular profiling of EVs, enabling unbiased identification of their lipidomic, proteomic, and metabolomic components. With excellent sensitivity and resolution, MS can uncover molecular heterogeneity among different EV subpopulations and link distinct physical fractions to specific molecular signatures, thereby explaining their functional diversity [286]. Commonly used MS-based platforms such as liquid chromatography–mass spectrometry (LC–MS) and matrix-assisted laser desorption/ionization mass spectrometry (MALDI-MS) are extensively applied for the characterization of EV lipids [287], proteins, and metabolites. Quantitative accuracy is further improved using isotopic labeling or label-free quantification, which enables precise comparative analysis of target molecules. Recent innovations have advanced MS applications in EV studies. Exosome-proximity ligation assay (Exo-PLA) coupled with high-resolution mass spectrometry (HRMS) enables discovery and validation of novel EV surface proteins [288], while charge detection mass spectrometry (CDMS) analyzes charge-to-mass ratios to reveal heterogeneity among medium-sized EVs [289]. Similarly, intact extracellular vesicle crosslinking mass spectrometry (iEVXL) technology has been used to characterize protein complex conformations within EVs [290]. Together, these advances have reinforced MS as a powerful tool for revealing the molecular complexity and biological functions of EVs [291].

Despite its remarkable analytical power, MS faces challenges in EV research. It requires specialized equipment, skilled operation, and highly pure EV samples for optimal accuracy [292]. In addition, low-abundance molecules and complex datasets pose interpretation challenges. However, emerging approaches such as single-EV mass spectrometry imaging and multi-omics data integration are expected to enhance sensitivity and expand the clinical applications of MS [293]. In the long term, systematic MS-based workflows will be instrumental in uncovering EV heterogeneity and advancing EV-based liquid biopsies and targeted therapeutic applications [294].

## Engineering strategies for next-gen therapeutic EVs

**Fig. 4:**
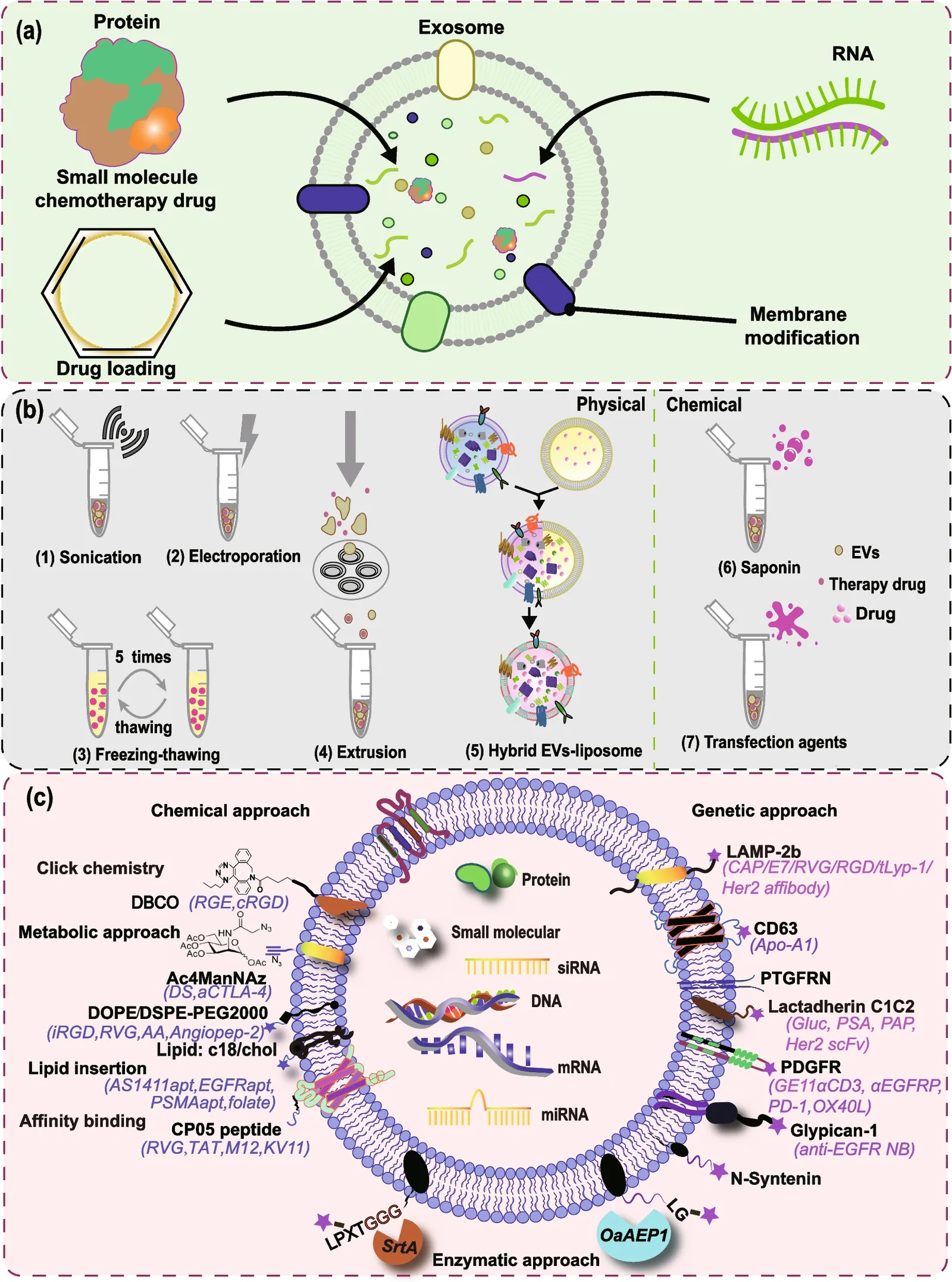
Engineering strategies for EVs.** a** Illustration of EV loading with different proteins, small molecule, or RNAs. **b** Physical and chemical methods used for cargo loading into EVs. Physical methods include (1) sonication, (2) electroporation, (3) freeze–thaw cycling, (4) extrusion, and (5) EV–liposome hybridization. Chemical methods include (6) saponin-mediated membrane permeabilization and (7) the use of transfection reagents. **c** EV surface functionalization using chemical, genetic, and enzymatic approaches. Chemical approaches include click chemistry, metabolic labeling, lipid insertion, and affinity-peptide binding. Genetic engineering enables the display of targeting ligands through EV-associated proteins, including LAMP-2b, CD63, PTGFRN, lactadherin C1C2, PDGFR, glypican-1, and N-syntenin. Enzymatic approaches employ SrtA and OaAEP1 for site-specific surface conjugation

Engineering strategies for EVs aim to overcome the intrinsic limitations of EVs—such as low yield, rapid clearance, limited loading capacity, and poor targeting efficiency—while enhancing their specificity, therapeutic potential, and stability. Generally, there are two major approaches for engineering EVs: endogenous modification, where producer cells are genetically or chemically modified before vesicle biogenesis, and exogenous modification, in which EVs are altered after isolation (Fig. 4) [130]. Collectively, these approaches transform EVs from passive biological carriers into programmable nanoplatforms with customized physicochemical and functional properties.

In endogenous engineering, the parental cells are modified to influence both the surface characteristics and cargo composition of the secreted EVs. These changes can be achieved by transducing or transfecting cells with specific plasmids or nucleic acids, which results in the selective packaging of therapeutic proteins or RNAs into the secreted EVs. The incorporation of RNA-binding motifs and sequence tags further improves targeted RNA loading during EV formation [295]. Additionally, genetic modification of membrane scaffolds such as tetraspanins, Lamp2b, or GPI-anchored proteins allows controlled surface display of peptides, antibodies, or reporter molecules that enhance receptor-mediated uptake or enable real-time tracking [130]. For example, Lamp2b-fused peptides have been reported for neuron-specific delivery [296]. Tetraspanin engineering supports stable ligand display without disrupting vesicle integrity [297]. Moreover, metabolic labeling of parental cells with azido-sugar analogs such as Ac₄ManNAz or Ac₄GalNAz introduces chemical handles on EV membranes that can be conjugated later with fluorescent dyes or targeting ligands through bio-orthogonal click reactions, enabling visualization or secondary modification without damaging EV structure [298, 299].

Alternatively, exogenous modification or engineering of EVs refers to post-isolation modifications. In this technique, the purified EVs from samples are directly used to incorporate therapeutic agents or surface ligands. To incorporate therapeutic agents into EVs, two main approaches are accomplished by active loading and passive loading [300]. The passive loading methods rely on simple incubation of EVs with the incorporating agents, which enables the hydrophobic molecules to integrate into the lipid bilayer. Active loading is achieved using different mechanical methods such as freeze–thaw cycles, sonication, extrusion, saponin permeabilization, and electroporation. Mechanistically, these methods help disrupt the membrane of EVs and enhance the encapsulation of hydrophilic and macromolecular cargoes. Optimization of such conditions is critical to maintain the morphology and biological activity of EVs [10]. To modify the surface of EVs after isolation for better internalization or attachment to the targeted site, EVs can be surface functionalized with antibodies, peptides, glycans, or aptamers via non-covalent interactions or chemical coupling. For instance, CD63-binding peptides and low-density lipoprotein (LDL)-mimetic constructs result in improved receptor-mediated internalization, while click chemistry enables covalent attachment of targeting ligands with high precision [301–303]. Moreover, tracking the in vivo fate of engineered EVs is essential for understanding their therapeutic outcomes and biodistribution. For this purpose, genetically encoded fluorescent or bioluminescent reporters like GFP-CD63 [304], NanoLuc-Lactadherin, or Gaussia luciferase fusions are used to enable noninvasive optical monitoring of EV trafficking [305, 306]. Alternatively, EVs labeled with radioactive isotopes allow high-sensitivity SPECT/PET imaging, which provides quantitative organ-level data and preserves EV integrity when appropriate chelation chemistry is used [307].

Overall, EV engineering integrates cellular, physical, and chemical modification strategies to modify EVs for efficient drug loading, targeted delivery, and precise in vivo tracking. By aligning the choice of parental scaffold, cargo loading method, and surface modification chemistry with the intended therapeutic goal, engineered EVs can achieve greater potency, specificity, and biocompatibility. These advancements position engineered EVs as a promising next-gen platform for diagnostics, drug delivery, and regenerative medicine.

## Applications of EVs in next‑gen drug delivery

EVs have emerged as a transformative platform in the field of cell-free therapeutics, which offers a biologically inspired alternative to cell-based therapies [96]. As natural nanocarriers of nucleic acids, proteins, and lipids, EVs mimic many beneficial effects of their parental cells and mitigate issues related to immune rejection, cell survival, and tumorigenicity. The inherent biocompatibility, stability, and capacity to cross biological barriers position EVs as powerful mediators of intercellular communication and promising vehicles for targeted therapeutic delivery. Consequently, EV-based therapy has gained considerable attention in regenerative medicine, oncology, and other clinical domains [308]. Mechanistically, the therapeutic efficacy of EVs is largely governed by their physicochemical and biological characteristics, which determine their in vivo fate and pharmacokinetic behavior. Characteristics such as morphology, particle size, membrane protein composition, and surface charge significantly influence their cellular uptake, biodistribution, and clearance profile [309]. It has been observed that smaller-sized EVs, such as exosomes, exhibit superior tissue penetration and longer circulation times [310]. The presence of specific surface ligands like tetraspanins (CD9, CD63) and integrins improves tropism toward target tissues or cells [311]. Moreover, surface charge modulates electrostatic interactions with the extracellular matrix and cell membranes, thereby affecting EV internalization [312]. Advances in molecular engineering and biofunctionalization enable the fine-tuning of these physicochemical parameters, improve targeting precision, controlled release, and therapeutic performance.

In regenerative medicine, EVs derived from MSCs demonstrate a remarkable capacity to promote tissue repair and functional recovery. For example, MSC-EVs suppress inflammation, facilitate angiogenesis, and inhibit apoptosis, thereby contributing to myocardial repair following ischemic injury [313]. Their paracrine actions also enhance cardiac remodeling and recovery post-myocardial infarction [314]. Furthermore, MSC-EVs play a pivotal role in neural regeneration, where they transfer neuroprotective proteins and RNAs that sustain neuronal viability and attenuate neuroinflammatory cascades in neurodegenerative disorders such as Alzheimer’s and Parkinson’s disease [315]. In addition, EVs hold immense promise in oncology treatment, where they can be utilized both as therapeutic targets and as intelligent drug-delivery systems. TD-EVs are known to mediate oncogenic signaling, immune evasion, and metastasis. Inhibiting TD-EV biogenesis, secretion, or uptake represents a novel approach to suppress tumor progression [316]. Conversely, engineered EVs can be repurposed as biocompatible nanocarriers for the targeted delivery of immunomodulators, chemotherapeutics, or RNA-based therapeutics, ensuring increased specificity and reduced systemic toxicity [317]. Their intrinsic tropism toward parental tumor cells and modifiable membrane properties allow the design of highly selective, ligand-decorated EVs for precision oncology. Early-phase clinical studies using autologous DC-derived EVs have demonstrated enhanced antigen presentation and activation of anti-tumor immunity, which highlights their potential in cancer immunotherapy [318].

### EVs as versatile platforms for drug delivery

EVs derived from distinct cell types exhibit characteristic surface proteins and nucleic acid composition, which define their targeting specificity and intercellular communication patterns [319, 320]. A primary advantage of EVs lies in their natural stability and barrier-crossing capabilities. The specialized surface molecules present on EV membranes can suppress immune activation by preventing interactions with antibodies and coagulation factors, thereby enhancing systemic compatibility and circulation time [321]. Moreover, EVs are inherently resistant to macrophage-mediated phagocytosis and lysosomal degradation, enabling them to remain stable in circulation and deliver therapeutic cargoes efficiently across biological barriers [322]. These properties provide EVs a unique advantage in sustained and site-specific drug delivery.

Another key benefit lies in the engineering flexibility of EVs. Through biological modification or hybridization with synthetic materials, EVs can be precisely tailored for enhanced therapeutic performance. Engineered EVs can be functionalized with targeting peptides, ligands, or polymers to enhance cellular uptake, tissue specificity, and intracellular accumulation of therapeutic agents [323].

This engineering versatility is particularly valuable for the delivery of nucleic acid therapeutics, which face unique biological barriers. Indeed, EVs are rapidly emerging as superior vectors for RNA-based therapeutics. Although modalities such as miRNAs, small interfering RNAs (siRNAs), and mRNAs represent transformative strategies for gene-based therapeutics [324], their clinical potential is frequently restricted by instability in biological fluids, rapid degradation by nucleases, and poor cellular permeability. Naked nucleic acids are readily cleared from circulation and may induce immune responses that reduce therapeutic efficacy [325]. In this context, EVs have gained recognition as biologically compatible delivery systems and address the challenges effectively. Their endogenous origin, nanoscale lipid bilayer, and natural ability to transport nucleic acids enable them to protect genetic material from degradation, improve intracellular delivery, and minimize immunogenicity, making them promising vectors for RNA-based therapeutics [326].

Collectively, the engineering flexibility and natural biocompatibility of EVs position them as a versatile and promising platform for drug delivery, with the potential to overcome many limitations of conventional synthetic carriers and accelerate the clinical translation of nucleic acid and small-molecule therapeutics. A summary of EV-mediated delivery of various therapeutic cargoes is provided in Table 3.

**Table 3:** EVs-mediated cargo delivery strategies for therapeutic applications

EV source/platform	Cargo type	Delivered cargo	Disease/condition	Loading/engineering strategy	Therapeutic outcome	Quantitative outcome	Reference
MSCs-derived EVs from PTX-primed SR4987 cells	Small molecule	PTX	Pancreatic cancer	Donor-cell PTX priming; EV-associated release	Inhibited pancreatic cancer cell proliferation in vitro	PTX-equivalent IC50: 2.73 ng/mL	[327]
HEK-293T-derived EVs	Combination cargo	PTX + ICG + SBC	Breast cancer	DMSO-assisted passive loading; pH/US-responsive release	Enhanced PA imaging-guided chemo-sonodynamic therapy	Cell viability reduced to 9.9% with US	[328]
Mesothelin CAR-T cells-derived EVs	Small molecule	PTX	Orthotopic lung cancer	CAR-engineered donor cells; PTX loading; inhalation delivery	Reduced lung tumor burden and systemic toxicity	CD8⁺ T-cell infiltration increased to 42.5%	[329]
Tumor-cells-derived EVs	Small molecule	DOX	Breast and ovarian cancers	Electroporation-mediated DOX loading	Increased antitumor efficacy and reduced cardiotoxicity	-	[330]
Cx43-positive HEK-293-cells-derived EVs	Small molecule	DOX	Breast cancer	Electroporation-mediated DOX loading	Enhanced antitumor activity with reduced cardiotoxicity	-	[331]
Grapefruits-derived EVs patched with heparin nanoparticles	Combination cargo	DOX-loaded heparin nanoparticles	Glioblastoma	Surface patching of DOX-loaded nanoparticles onto EVs	Improved drug delivery to brain tumors and prolonged survival	Median survival increased to 41 days	[332]
HSSP-modified BMSC-derived EVs	Combination cargo	TMZ + STAT3 siRNA	TMZ-resistant glioblastoma	HSSP surface modification with TMZ/siRNA loading	Overcame TMZ resistance and enhanced glioblastoma treatment	-	[333]
PDGFA-ligand-modified NSC-derived EVs	hormone	T3	Demyelination/remyelination	Donor-cell PDGFA engineering; T3 loading into targeted EVs	Enhanced OPC targeting and reduced neuroinflammation	Significantly promoted remyelination compared with free T3	[334]
Fe65-engineered neuronal EVs	Small molecule	Corynoxine-B	Alzheimer’s disease	Donor-cell Fe65 engineering; Corynoxine-B encapsulation	APP-targeted delivery, autophagy induction, and amyloid clearance	Ameliorated cognitive decline and AD pathology	[335]
EL-4 cells-derived EVs	Small molecule	Curcumin	Septic shock/systemic inflammation	Curcumin complexation with EVs	Improved anti-inflammatory activity and protection against LPS-induced septic shock	Survival remained ~75% at day 4	[336]
Microglia-derived EVs	Combination cargo	Berberine + palmatine	Alzheimer’s disease	Co-loading of berberine and palmatine into EVs	Improved BBB delivery and regulated neuroinflammation in AD	Improved learning/memory and reduced Aβ pathology	[337]
MSC-derived EVs	miRNA	miR-146b	Glioma	Donor-cell miR-146b plasmid transfection; EV-mediated miRNA delivery	Suppressed EGFR/NF-κB signaling in glioma cells	Significantly reduced glioma xenograft growth	[338]
GBM-derived EVs	miRNA	miR-1	Glioblastoma	miR-1 loading/restoration in GBM-derived EVs	Reprogrammed EV-mediated tumor microenvironment signaling	Reduced GBM tumorigenicity, angiogenesis, invasion, and neurosphere formation	[339]
GE11-modified HEK-293-cells-derived EVs	miRNA	let-7a	EGFR-expressing breast cancer	Donor-cell GE11 surface engineering; let-7a loading	Targeted EGFR-positive breast cancer cells and delivered antitumor miRNA	Markedly suppressed tumor growth	[340]
ECFC-derived EVs	miRNA	miR-486-5p	Acute kidney injury	Endogenous miR-486-5p-enriched EVs; systemic infusion	Activated PTEN/Akt signaling and protected against ischemic kidney injury	Significantly reduced creatinine, BUN, neutrophil infiltration, and injury score	[341]
RVG29-modified hucMSC-derived EVs	miRNA	miR-133b	Parkinson’s disease	RVG29 surface modification; Exo-Fect-mediated miR-133b loading	Brain-targeted delivery and RhoA/ROCK/Tau pathway modulation	Improved motor function and attenuated 6-OHDA-induced nerve damage	[342]
RVG-Lamp2b-modified HEK-293-cells-derived EVs	miRNA	miR-25 or miR-181a	Spinocerebellar ataxia type 3	RVG-Lamp2b donor-cell engineering; electroporation-mediated miRNA loading	Reduced mutant ATXN3 expression and neuronal loss	Rotarod latency increased to 221.79 s with miR-25 and 200.63 s with miR-181a vs 130.13 s in SCA3 control	[343]
CD47-SST-engineered dendritic cell-derived EVs	miRNA	miR-29b-2	Alzheimer’s disease	Donor-cell CD47/SST engineering with miR-29b-2 enrichment	Enhanced brain delivery and PSEN1 silencing	Reduced β-amyloid oligomer production	[344]
BMSC-derived EVs	miRNA	miR-25	Fracture healing	Endogenous miR-25-enriched EVs; local delivery to fracture site	Enhanced osteoblast differentiation, proliferation, and migration	Significantly promoted fracture healing in mice	[345]
NGF-preconditioned MSC-derived EV-functionalized scaffold	miRNA	NGF-elicited exosomal miRNAs	Innervated bone regeneration	NGF donor-cell preconditioning; EV loading onto 3D-printed porous scaffold	Promoted neurovascular structure formation and bone regeneration	Significantly increased BV/TV (38.7%), BMD, vessel area, and nerve area	[346]
CAP-modified dendritic cell-derived EVs	miRNA	miR-140	Osteoarthritis	CAP-Lamp2b donor-cell engineering; miR-140 loading; intra-articular delivery	Targeted chondrocytes and inhibited cartilage-degrading enzymes	OARSI score reached a level similar to sham control	[347]
IPFP-MSC-derived EVs	miRNA	miR-100-5p	Osteoarthritis	Endogenous miR-100-5p-enriched EVs; intra-articular delivery	Protected articular cartilage by inhibiting mTOR and enhancing autophagy	Significantly reduced OA severity and improved gait abnormalities	[348]
Inflammation-stimulated ADMSC-derived EVs	miRNA	miR-27b-3p	TMJ osteochondral defect	Inflammatory donor-cell stimulation; EV-functionalized scaffold delivery	Promoted macrophage M2 polarization and TMJ condylar regeneration	BV/TV increased to 79.201 ± 2.124% at 8 weeks	[349]
Exosome-liposome hybrid EV-like nanovesicles	Combination cargo	siBACE1 + TREM2 plasmid	Alzheimer’s disease	Angiopep-2/ROS-responsive hybrid nanovesicle co-delivery	Reduced Aβ production and reprogrammed microglia toward an anti-inflammatory phenotype	Improved cognitive function	[350]
MSC-derived EVs	siRNA	FTO siRNA	Parkinson’s disease	si-FTO loading into MSC-derived EVs	Reduced dopaminergic neuronal death through m6A/ATM regulation	Suppressed α-Syn and neuronal death while restoring TH expression	[351]
Genetically engineered HEK-293T-cells-derived EVs	mRNA	CD-UPRT mRNA	Schwannoma	Donor-cell CD-UPRT engineering; intratumoral EV delivery with systemic 5-FC	Prodrug-activated suicide gene therapy inhibited tumor growth	Significantly inhibited schwannoma tumor growth in vivo	[352]
ARRDC1- or CD63-engineered HEK-293T-cells-derived EVs	mRNA	p53 mRNA	p53-null cancer cells	Donor-cell ARRDC1–p53 or CD63–p53 fusion engineering	Restored p53 delivery and induced tumor-suppressive activity	Significantly increased apoptosis in H1299 cells	[353]
Oligodendrocyte-derived EVs	mRNA	SIRT2	Major depressive disorder	Endogenous SIRT2-containing EVs; systemic delivery	Restored hippocampal neurogenesis and synaptic plasticity	-	[354]
RVG-engineered HEK-293-cells-derived EVs	mRNA	NGF mRNA	Cerebral ischemia	Donor-cell RVG-Lamp2b and NGF co-engineering; systemic delivery	Reduced neuroinflammation, promoted cell survival, and enhanced neurogenesis	M1 microglia reduced to 39.4%; M2 microglia increased to 67.5%	[355]
Arc-engineered leukocyte-derived EVs	mRNA	Reporter mRNA	Neuroinflammation-associated neuronal delivery	Arc capsid/A5U engineering for enhanced mRNA packaging and systemic delivery	Enhanced BBB crossing and neuronal mRNA delivery in neuro-inflammatory sites	-	[356]

#### EVs-mediated small molecule delivery

A large proportion of conventional chemical drugs suffer from poor solubility, rapid systemic clearance, and dose-limiting toxicity. These drawbacks not only curtail therapeutic efficacy and clinical applicability but also result in poor absorption, low bioavailability, and severe off-target toxicity. EV-based delivery has emerged as an excellent platform to overcome these hindrances. By improving solubility, extending circulation time, and enabling cell-specific targeting, EVs can circumvent the major pharmacokinetic and safety barriers associated with conventional drug formulations.

**Fig. 5:**
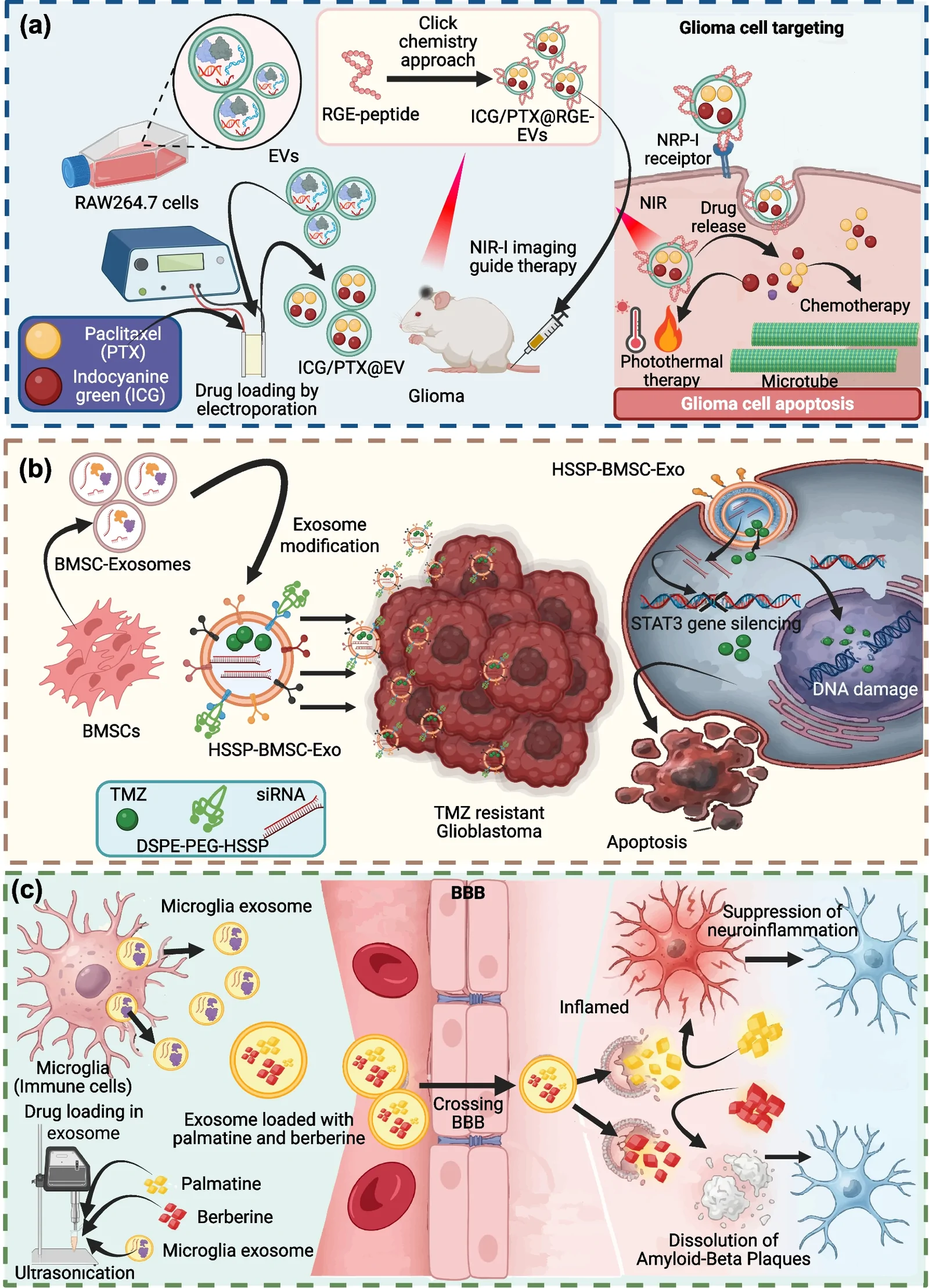
EVs as versatile platforms for drug delivery. **a** Schematic illustration of RGE-modified biomimetic exosomes (ICG/PTX@RGE-EVs) for targeted glioma therapy; Macrophage-derived EVs are loaded with Paclitaxel (PTX) and Indocyanine Green (ICG), and surface-functionalized with RGE peptides. The RGE peptide specifically binds to Neuropilin-1 (NRP-1) receptors overexpressed on glioma cells, facilitating internalization. Upon 808 nm NIR laser irradiation, ICG generates local hyperthermia (Photothermal therapy), which triggers the rapid release of PTX, resulting in synergistic apoptotic cell death. **b** Schematic representation of HSSP-engineered exosomes co-delivering siSTAT3 and TMZ to overcome glioblastoma drug resistance; Preparation of DSPE-PEG-HSSP modified exosomes loaded with therapeutic cargo and its specific targeting of HMOX1 receptors on resistant GBM cells. The intracellular release of HSSP-BMSc-Exo lead to STAT3 silencing and re-sensitization to TMZ, triggering apoptosis. **c** Schematic mechanism of Microglia exosomes co-delivering Berberine and Palmatine for AD therapy; Microglia exosomes are isolated from microglia and loaded with Berberine and Palmatine. The exosomes leverage their natural immune-homing ability to cross the BBB. Upon reaching the brain parenchyma, Microglia exosomes target sites of neuroinflammation, releasing their cargo to inhibit pro-inflammatory cytokines, promote M2 microglial polarization, and reduce Amyloid-β Aβ plaque deposition. Figures were created in BioRender. https://BioRender.com/tgeoptt

The integration of EVs into chemotherapeutic delivery systems has been demonstrated in several innovative studies. For instance, PTX, a potent chemotherapeutic agent widely used in lung cancer treatment, demonstrates strong antitumor activity but is constrained by severe systemic toxicity [357]. In a biomimetic approach, MSCs were primed with PTX, allowing the cells to uptake and repackage the drug into secreted EVs. These PTX-associated EVs effectively delivered chemotherapy to pancreatic cancer cells and inhibited their proliferation, with a PTX-equivalent IC50 of 2.73 ng/mL [327]. In a separate strategy to enhance precision, macrophage-derived EVs were loaded with PTX and their surfaces were modified with RGE peptides to target the neuropilin-1 (NRP-1) receptor. The resulting construct, ICG/PTX@RGE-EV, achieved efficient glioma targeting (Fig. 5a). Furthermore, the inclusion of pH-responsive components such as sodium bicarbonate facilitated drug release within the acidic microenvironment of lysosomes, thereby boosting intracellular bioavailability [328, 358]. In another study, researchers developed a targeted delivery platform using EVs derived from mesothelin (MSLN)-specific Chimeric antigen receptors T (CAR-T) cells. These engineered EVs were loaded with PTX and administered via inhalation to directly target orthotopic lung tumors. This non-invasive delivery route allowed the drug to accumulate specifically within the tumor tissue, which effectively suppressed tumor growth and minimized damage to the liver and kidneys. Furthermore, the treatment successfully reversed the immunosuppressive nature of the tumor microenvironment, a change driven by the increased infiltration of CD8^+^ T cells and the release of anti-tumor cytokines [329].

EVs also offer a strategy to mitigate the toxicity of established drugs. DOX, a cornerstone chemotherapeutic agent with potent antitumor activity but causing serious cardiotoxicity, has been reformulated by using EV carriers to reduce cardiac side effects. EVs encapsulated DOX demonstrated reduced cardiotoxicity with strong anticancer potency [330, 331]. In a recent study, Niu et al. employed a novel approach by using grapefruit-derived EVs as a surface-display platform. Rather than encapsulating the drug, they patched DOX-loaded heparin nanoparticles onto the surface of the PDEVs. This biomimetic strategy resulted in a four-fold increase in drug payload compared to conventional methods and enabled efficient crossing of the BBB, along with deep tumor penetration, and in vivo treatment prolonged median survival to 41 days, as well as offer a scalable solution for glioma treatment [332]. On the other hand, in the case of temozolomide (TMZ), drug resistance in glioblastoma (GBM) is predominantly driven by the overexpression of O(6)-methylguanine-DNA methyltransferase (MGMT), which repairs TMZ-induced DNA damage [359]. To counteract this resistance, researchers engineered bone marrow mesenchymal stem cell derived EVs (BMSC-EVs) to carry a peptide targeting heme oxygenase-1 and co-deliver TMZ and STAT3-targeting siRNA. Notably, STAT3 is a critical upstream activator of MGMT transcription; therefore, silencing STAT3 effectively downregulated MGMT expression and re-sensitized GBM cells to TMZ (Fig. 5b) [333]. Such approaches highlight the ability of EVs to circumvent cellular defense mechanisms and improve chemotherapeutic responsiveness.

EV-mediated drug delivery has also become a rapidly expanding field in neurotherapeutics, offering an effective strategy for transporting small molecules across the BBB with high precision and minimal immunogenicity. In Alzheimer’s disease (AD), several EV-based systems have been developed to tackle two distinct pathological hallmarks: demyelination, and proteopathic accumulation. To repair the damaged myelin sheath, engineered NSCs-EVs were modified to display the PDGFA ligand for targeted delivery to oligodendrocyte progenitor cells (OPCs) within demyelinated lesions. When loaded with the thyroid hormone T3 (3,5,3′-triiodothyronine), these modified EVs demonstrated superior efficacy compared to free drug administration in a cuprizone-induced injury model. The treatment not only significantly enhanced remyelination but also restored the pathological microenvironment by reducing astrogliosis and microglial activation [334]. To clear proteopathic aggregates, Fe65 overexpressed neuronal EVs were engineered to encapsulate Corynoxine-B, which induced autophagy in amyloid precursor protein (APP)-overexpressing neurons, facilitated amyloid clearance, and restored cognitive performance in transgenic AD mice [335].

Besides synthetic compounds, EV-mediated delivery has also been applied to natural bioactive compounds. Medicinal compounds such as curcumin, a plant-derived compound with anti-tumor and anti-inflammatory properties, have also been explored. The therapeutic efficacy of curcumin is hindered by its poor solubility and rapid metabolism. To overcome this problem, researchers encapsulated curcumin within EL-4 cells-derived EVs. This biological nanocarrier system notably enhanced the drug's solubility and prolonged its circulation time. More importantly, it facilitated targeted delivery to activated myeloid cells, significantly improving therapeutic efficacy in septic shock while minimizing the systemic toxicity typically noticed with free curcumin administration [336]. Furthermore, a recent study utilized microglial EVs to co-deliver berberine and palmatine, which are plant-derived isoquinoline alkaloids. This dual-drug system showed enhanced BBB penetration compared to single-drug treatments. In amyloid precursor protein/presenilin-1 (APP/PSEN1) transgenic mice, the combined therapy produced a synergistic effect by modulating inflammatory cytokine release and inhibited amyloid-β plaque formation, ultimately reversing cognitive deficits related to memory and learning (Fig. 5c) [337]. These examples collectively demonstrate that EVs can serve as efficient and biocompatible nanocarriers for diverse therapeutic agents.

#### EVs-mediated miRNA delivery

Small non-coding RNAs such as miRNAs and siRNAs represent a transformative strategy for gene silencing therapies in modern medicine. However, their clinical potential is frequently restricted by instability in biological fluids, rapid degradation by nucleases, and poor cellular permeability. EVs effectively overcome these challenges by shielding these cargoes within their lipid bilayer and enabling efficient intracellular delivery.

Among the various genetic cargos carried by EVs, miRNAs are the most extensively studied. In oncology, MSC-EVs enriched with miR-146b-5p could reduce glioma invasiveness by binding to epidermal growth factor receptor (EGFR) mRNA, which leads to the suppression of oncogenic pathways [338]. Another report found that EVs carrying miR-1 inhibited proliferation, angiogenesis, and invasion in glioblastoma models and demonstrated their potential in antitumor therapy [339], while EVs containing Let-7 targeted EGFR suppressed breast cancer progression [340]. Beyond cancer, EV-mediated miRNA transfer has shown therapeutic value in non-tumor contexts. For instance, miR-486-5p-loaded EVs were shown to attenuate acute renal injury [341].

In the nervous system, RVG29 peptide-modified EVs encapsulating miR-133b, improved motor coordination, enhanced dopaminergic function, and reduced Tau phosphorylation by modulating the RhoA/ROCK pathway [342]. Moreover, the RVG-engineered EV platform enabled the combined delivery of miR-25 and miR-181a for treating spinocerebellar ataxia type 3 (SCA3), a progressive neurodegenerative disorder driven by an expanded CAG repeat in the ataxin 3 (ATXN3) gene. This genetic abnormality results in toxic ATXN3 aggregates, Purkinje neuron degeneration, and pronounced motor dysfunction. With the RVG modification, the engineered EVs successfully crossed the BBB and selectively delivered both miRNAs into targeted neuronal populations. In SCA3 mouse models, this targeted delivery strategy substantially reduced mutant ATXN3 levels, prevented neuronal apoptosis, and mitigated key neuropathological changes. Consequently, motor coordination was significantly enhanced, as reflected by a rotarod latency increasing to 221.79 s after miR-25 delivery and 200.63 s after miR-181a delivery, compared with 130.13 s in SCA3 control mice [343]. On the other hand, in AD engineered DC-EVs encapsulating miR-29b-2 effectively reduced PSEN1 expression and decreased β-amyloid accumulation both in vitro and in vivo. The modified EVs, functionalized with CD47-somatostatin (SST), targeted the hippocampus after intravenous injection and significantly lowered Aβ1–42 oligomer levels in transgenic AD mice, thereby leading to improved cognitive performance [344].

In bone defect repair and regeneration EV-delivered miRNAs play an increasingly recognized role. In a recent study, in vivo delivery of EVs-miR-19b promoted bone healing, as evidenced by a 35% increase in callus area and a 50% enhancement in mineralized callus formation within four weeks [345]. Because vascularization is critical for bone repair, EVs carrying angiogenic regulatory miRNAs have also been explored. TREMD-Exos loaded with miR-142-3p enhanced osteogenesis and angiogenesis by increasing bone volume/total volume (BV/TV) by 40% and vascular density by 1.9-fold in treated fractures [360]. To further advance bone repair, multifunctional EVs have been engineered to couple neurogenic and osteogenic regeneration. Pretreating adipose-derived mesenchymal stem cells (ADMSCs) with nerve growth factor (NGF) produced neurotrophic EVs rich in miR-195-5p, let-7a-5p, and miR-29b-3p. These EVs not only promoted neural recovery by enhancing Schwann cell migration and upregulating neurotrophins BDNF and neurotrophin-3 (NT-3), but also synergistically enhanced osteogenesis, as evidenced by a 5.7-fold increase in runt-related transcription factor 2 (RUNX2) and osteocalcin (OCN) levels, and a final BV/TV of 38.7% [346].

In the therapeutic landscape of osteoarthritis (OA), EVs can function as intrinsic gene vectors that regulate inflammation, matrix equilibrium, and cell survival. For example, modifying EVs with chondrocyte-affinity peptides allowed for the precise delivery of miR-140 to cartilage cells. This targeted approach significantly attenuated inflammatory mediators and catabolic enzymes and effectively retarding the progression of OA [347]. In another study, exosomes isolated from infrapatellar fat pad MSCs have been found to safeguard articular cartilage by balancing ECM metabolism and preventing cell death. Mechanistically, these protective effects rely on miR-100-5p, which targets the mammalian target of rapamycin (mTOR) pathway to upregulate autophagy [348]. Finally, EVs from ADMSCs have demonstrated regenerative potential in condylar osteochondral defects. While both standard and inflammation-primed exosomes support BMSC growth, the latter were enriched with miR-27b-3p. This specific miRNA targets colony stimulating factor 1(CSF-1) to induce M2 macrophage polarization, thereby fostering an immune environment conducive to tissue repair [349]. Collectively, these findings illustrate that EVs serve as natural and efficient carriers for miRNA-based therapeutics.

#### EVs-mediated siRNA delivery

Alongside miRNAs, siRNAs have emerged as powerful tools for targeted gene silencing. siRNAs induce sequence-specific mRNA degradation through RNA interference, enabling the selective suppression of disease-associated genes. However, due to their hydrophilicity and negative charge, naked siRNAs are unstable in serum, have short half-lives, and exhibit poor membrane permeability [361]. EVs effectively overcome these challenges by encapsulating siRNAs within their lipid membranes, shielding them from nucleases and promoting receptor-mediated uptake by target cells. Consequently, the therapeutic application of siRNA-loaded EVs has advanced considerably, supported by innovative loading strategies [362].

For instance, the "Shock Wave-mediated Extracellular Vesicle Engineering Technology" (SWEET) was utilized to load KRASG12C-targeting siRNA into bovine milk EVs, effectively silencing KRAS expression and suppressing tumor growth in non-small cell lung cancer (NSCLC) models [363]. Another study designed c(RGDyK)-modified EVs delivering siRNA against PD-L1, which restored anti-tumor immunity in glioblastoma following radiotherapy by increasing CD8⁺ T-cell infiltration into the tumor microenvironment [364]. Furthermore, exosomes derived from M1 macrophages were co-loaded with small interfering RNA targeting signal regulatory protein α (siSIRPα) and siSTAT6 to promote macrophage repolarization. These engineered vesicles successfully repolarized pro-tumor M2 macrophages back into the anti-tumor M1 phenotype, suppressed the migration and invasion of 4T1 breast cancer cells, and enhanced macrophage-mediated tumor cell phagocytosis [365]. In one investigation, MSC-EVs were surface-modified with urokinase plasminogen activator (uPA) and encapsulated with siSrc to create a dual-targeting complex. This system specifically homed in on both tumor cells and stromal cells that had entered a senescent state due to chemotherapy. When administered alongside DOX, this formulation significantly lowered the burden of senescent cells and improved therapeutic efficacy [366].

Beyond oncology, EV-enabled siRNA delivery has shown promise in neurological disease models, where crossing the BBB and achieving cell-selective uptake remain major challenges. Leveraging the homing capability of exosomes and the assistance of Ang2 peptides, a hybrid EV–lipid nanoparticle system co-delivering siBACE1 (targeting β-site APP cleavage enzyme 1) and a TREM2-encoding plasmid was demonstrated to effectively penetrate the BBB and promote drug accumulation at sites of AD pathology. The expressed TREM2 shifted microglia from a pro-inflammatory M1 toward an anti-inflammatory M2 phenotype, facilitating amyloid clearance while attenuating neuroinflammation. Concurrent siBACE1 delivery suppressed amyloid generation, thereby further synergistically potentiating the therapeutic efficacy in AD [350]. In Parkinson’s disease (PD), MSC-EVs carrying FTO-targeted siRNA (siFTO) efficiently delivered the cargo to the striatum of PD mice. Since FTO is a key upstream 'switch' in the pathological pathway leading to α-synuclein accumulation, siFTO significantly reduced α-synuclein expression, thereby alleviating dopaminergic neuronal death and restoring ataxia telangiectasia mutated gene balance [351].

In addition to mammalian EVs, OMVs have also been explored for siRNA delivery. In a proof-of-concept study focused on targeting the immune checkpoint, the PD-L1 bacterial OMV system demonstrated substantial effectiveness. In contrast to conventional regimens requiring repeated dosing, a single injection of the engineered bacteria achieved nearly 50% PD-L1 knockdown. This sustained immune checkpoint blockade, driven by the continuous release of siRNA from OMVs, yielded a tumor suppression rate of 49.4% in vivo. These results highlight the potential of using bacteria to produce RNA-loaded OMVs directly within the body, offering a more streamlined approach to tumor therapy without the complexities associated with pre-formulation processes [367]. Collectively, these studies highlight EVs as effective carriers that improve siRNA stability and delivery, enabling robust gene silencing across diverse disease models.

#### EVs-mediated mRNA delivery

Beyond small RNA molecules, mRNA-based therapeutics have gained attention for their ability to directly express functional proteins within target cells. Although lipid and polymeric nanocarriers have been extensively studied for mRNA delivery, challenges such as nonspecific biodistribution and unintended immune activation continue to hinder their effectiveness [368]. In contrast, EVs, as natural carriers of mRNA, present a compelling alternative due to their superior biocompatibility and ability to target specific sites more precisely [326, 369, 370]. A study demonstrated that EVs encapsulating cytosine deaminase–uracil phosphoribosyltransferase (CD-UPRT) mRNA efficiently converted the prodrug 5-fluorocytosine to 5-fluorouracil at tumor sites, thereby achieving localized tumor inhibition [352]. Another investigation developed inhalable EVs carrying IL-12 mRNA for pulmonary delivery. Once internalized, IL-12 mRNA stimulated interferon-gamma (IFN-γ) production and reprogrammed the tumor microenvironment (TME) to enhance immune activation and antitumor effects [371].

Recent advancements in EV engineering have further enabled the delivery of genetic payloads designed to induce cell death in resistant tumors. In one approach targeting HER2-positive breast cancer, EVs were encapsulated with mRNA encoding the N-terminal domain of gasdermin D (GSDMD-N). This strategy successfully facilitated intratumoral translation and triggering pyroptosis, which significantly hindered tumor growth [372]. Building on such targeted strategies, fusion constructs with vesicle-associated proteins such as arrestin domain dontaining 1 (ARRDC1) and CD63, enhanced the incorporation of p53 mRNA and protein into EVs. These engineered EVs restored p53 function in p53-null H1299 lung cancer cells, inhibited proliferation, and increased apoptosis, with ARRDC1–p53 EVs increasing apoptosis to approximately 23.8% compared with approximately 9.7% in the control group [353].

Beyond oncology, EV-mediated gene delivery has also shown promise in central nervous system (CNS)-related conditions. In major depressive disorder (MDD), oligodendrocyte-derived EVs enriched with sirtuin 2 (SIRT2) reversed depression-like behaviors in mice exposed to chronic unpredictable mild stress. Mechanistically, the EVs delivered SIRT2 into neural cells, where it deacetylated protein kinase B (AKT) and activated the AKT/glycogen synthase kinase 3β (GSK-3β) pathway, thereby enhancing hippocampal neurogenesis and restoring synaptic plasticity. The essential role of SIRT2 was confirmed by the loss of antidepressant effects upon SIRT2 inhibition. Complementary in vitro experiments further showed that SIRT2-rich EVs boosted neuronal differentiation and upregulated synaptic protein expression [354]. Moreover, mRNA-loaded EVs have been explored for CNS regeneration. RVG-engineered EVs carrying nerve growth factor (NGF) mRNA successfully reached the ischemic cortex following systemic administration and promoted neuronal survival and recovery in ischemic brain models [355]. Additionally, leukocyte-derived EVs containing retrovirus-like capsids (Arc) efficiently packaged mRNA and crossed the BBB by leveraging endothelial adhesion molecules, which enable targeted delivery to inflamed brain regions [356].

The nucleic acid cargos within EVs not only reflect the physiological state of their parental cells but can also actively influence recipient-cell behavior. Upon delivery, EV-associated mRNAs can be translated into functional proteins that reshape downstream signaling pathways and modulate cellular responses [373]. Overall, compared with traditional synthetic delivery systems, EVs provide a biocompatible, stable, and versatile platform for transporting therapeutic nucleic acids and other biomacromolecules. Their engineerable surfaces enable improved targeting and cellular uptake, supporting more efficient and potentially safer RNA and mRNA-based therapies [374]. Continued advances in EV engineering, cargo-loading strategies, and hybrid nanotechnologies are expected to accelerate clinical translation and further establish EVs as next-gen carriers for gene-based therapeutics.

### EVs in cancer vaccination and immunotherapy

EV-based vaccines have emerged as appealing platforms for cancer vaccination and immunotherapy because of their natural origin, biological adaptability, and suitability for bioengineering. Compared with live cell-based vaccines, EVs offer practical advantages such as improved safety, easier storage, greater experimental control, and better scalability for clinical translation. In contrast to many conventional nanotherapeutic platforms, including liposomes, polymeric nanoparticles, lipid nanoparticles, and viral vectors, EVs also possess intrinsic biological features that are difficult to reproduce synthetically, such as parental-cell-derived membrane proteins, immune-regulatory cargo, natural tissue tropism, low immunogenicity, and the capacity to participate in intercellular immune communication [375]. This is because EVs natively carry a repertoire of immunologically active molecules—including MHC molecules, co-stimulatory ligands, and regulatory proteins—that enable them to directly engage immune cells. Therefore, in cancer vaccine and immunotherapy applications, EVs should not be viewed only as passive cargo carriers; rather, they can act as biologically active immune-communication vesicles that integrate antigen delivery, antigen presentation, immune-cell targeting, and immune activation within a single nanoscale system.

While DC-EVs and OMVs also hold promise for antiviral and antibacterial vaccine development, those applications are discussed separately in the next section. Here, we focus on their roles in cancer immunotherapy. This distinctive immunological function is closely related to the source-dependent composition of EVs. TD-EVs can contribute to immune escape and metastasis; however, when appropriately engineered or purified, they can also serve as rich reservoirs of tumor-associated antigens (TAAs) and neoantigens for antitumor vaccination [11, 376]. These tumor antigens can be taken up and presented by dendritic cells, thereby activating tumor-specific cytotoxic T lymphocytes and promoting antitumor immunity [377, 378]. Conversely, DC-EVs naturally display MHC class I and II molecules, peptide-MHC complexes, co-stimulatory molecules, and immunoregulatory signals, enabling direct or indirect activation of both CD8⁺ cytotoxic T cells and CD4⁺ helper T cells [379, 380]. Additionally, EVs derived from macrophages [381], B cells [382], and T cells [383] exhibit source-specific immunostimulatory properties that can be exploited for antigen transfer, immune-cell activation, or tumor microenvironment remodeling. Furthermore, OMVs can also be exploited in cancer immunotherapy because their pathogen-associated molecular patterns promote antigen-presenting cell activation, IFN-γ release, and antigen-specific T-cell responses [384, 385]. Although DC-EVs and OMVs are also important for antiviral and antibacterial vaccine development, those infectious disease applications are discussed in detail in the next section.

Preclinical investigations further support the advantage of EV-based vaccines over selected synthetic nanocarriers. Li et al*.* [386] directly compared DC-EVs neoantigen vaccines with liposome-based neoantigen formulations in MC-38 colon carcinoma and B16F10 melanoma models. The exosome-based vaccine produced stronger antitumor immunity than the liposomal formulation, including improved tumor growth inhibition, enhanced CD4⁺ and CD8⁺ T-cell infiltration, prolonged survival, reduced lung metastasis, and durable antitumor memory. Importantly, when exosomal surface proteins were removed by proteinase K treatment, the antitumor effect became comparable to that of liposomes and weaker than intact exosome vaccines, indicating that EV-associated surface proteins substantially contribute to their superior immunotherapeutic activity. This finding provides direct evidence that EV vaccines can outperform liposomal systems not only because of cargo delivery, but also because of their biologically active membrane components.

Beyond functioning as standalone vaccines, EVs can also serve as immune-functional components that augment the performance of inorganic or hybrid nanotherapeutic systems. In a photo-nanovaccine strategy, serum exosomes from hyperthermia-treated tumor-bearing mice were used to encapsulate black phosphorus quantum dots, generating hEX@BP. Compared with black phosphorus quantum dots alone, hEX@BP showed improved tumor targeting, prolonged photothermal performance, stronger tumor heating, enhanced tumor suppression, Th1-type cytokine induction, and increased CD4⁺ and CD8⁺ T-cell infiltration [387]. This example suggests that EVs can add antigen-carrying, immune-stimulating, and tumor-homing functions to inorganic photothermal platforms. Similarly, photosensitive hybrid γδ-T exosomes prepared by fusing γδ-T-cell-derived exosomes with Ce6-loaded liposomes retained EV-associated functional proteins such as C–C chemokine receptor type 5 (CCR5), PD-1, MHC-I, MHC-II, and CD86, while also incorporating the photosensitizer cargo from liposomes. These hybrid EVs showed higher melanoma accumulation than Ce6-loaded liposomes, induced synergistic tumor cell killing through cytolytic molecules and ROS generation, promoted dendritic-cell maturation, and stimulated melanoma antigen-specific CD4⁺ and CD8⁺ T-cell responses [388]. Thus, EVs can be further exploited not only as standalone vaccines but also as immune-functional components of hybrid nanotherapeutic systems.

Despite these advantages, the inherent complexity of EV-based vaccines—in terms of heterogeneous antigen loading, variable MHC-peptide presentation, and mixed co-stimulatory/inhibitory signals—necessitates rigorous preclinical and clinical optimization. The immunological properties of EVs can be further enhanced by rational engineering. Current strategies include loading EVs with defined tumor antigens or neoantigens, increasing MHC-peptide display, decorating EV membranes with targeting ligands, incorporating adjuvants such as TLR agonists, and genetically modifying parental cells that enriching immunostimulatory molecules while reducing immunosuppressive cargo [298, 389, 390]. EVs can also be combined with checkpoint blockade or engineered to modulate PD-1/PD-L1 and CD47/SIRPα signaling. These strategies collectively improve tumor recognition, antigen presentation, macrophage phagocytosis, and T-cell-mediated immunity [391, 392]. In addition, EV-mediated delivery of stimulator of interferon genes (STING) agonists, or immunogenic-cell-death-inducing chemotherapeutics can promote dendritic-cell maturation, Th1-biased immune activation, CD8⁺ T-cell recruitment, and durable antitumor responses [11, 393, 394]. Collectively, these strategies indicate that the main advantage of EV-based vaccines lies in their ability to combine antigen carriage, immune signaling, and therapeutic engineering in one biologically active platform***.***

#### Dendritic cell-derived EVs

DC-EVs play a major role in vaccine-based therapies. Early in vivo studies demonstrated that DC-EVs presenting TAAs bound to MHC class I molecules can elicit strong CD8⁺ T-cell activation, ultimately providing measurable protection against tumor growth in immunocompetent mice [395]. These findings positioned DC-EVs as not only efficient antigen carriers but also potent initiators of adaptive antitumor immunity. Mechanistically, one of the central mechanisms supporting their activity is the process known as “cross-dressing,” in which intact peptide–MHC complexes on DC-EVs membranes are directly transferred to recipient DCs, thereby bypassing the need for intracellular antigen processing and enabling rapid T-cell activation [396]. Beyond their role in adaptive immunity, DC-EVs also enhance innate responses, as they are enriched in immunostimulatory ligands such as natural killer group 2 member D (NKG2DLs) and IL-15Rα, which drive NK cell expansion and IFN-γ production [397].

The clinical potential of DC-EV-based vaccines was first evaluated in a phase II study involving patients with non–small cell lung cancer (NSCLC), where IFN-γ–matured DC-EVs (a maturation step that upregulates MHC and co-stimulatory molecules) loaded with MHC class I and II-restricted tumor peptides were shown to be safe, though their immunogenicity required further optimization [398]. Follow-up investigations improved upon this approach by incorporating full-length tumor antigens, which—by providing a broader repertoire of T-cell epitopes and enabling processing through both MHC class I and II pathways**—**more effectively activated both CD4⁺ and CD8⁺ T-cell responses compared with peptide-only formulations [399]. Additional advancements included a self-adjuvanted DC-EVs platform capable of co-delivering TAAs with poly (I:C), leading to pronounced tumor inhibition in melanoma models [400]. In a complementary engineering approach, another group utilized a surface-engineering technique termed “exosome painting” to conjugate the functional domain of the immunostimulatory adjuvant HMGN1 onto DC-EVs. This engineered EV-adjuvant complex markedly strengthened DC activation, improved their migration to lymphoid organs, and promoted the generation of long-lasting memory T-cell responses, demonstrating high efficacy in suppressing tumor growth in difficult-to-treat models such as orthotopic hepatocellular carcinoma [301]. More recent developments have moved toward personalized immunotherapy, where patient-specific neoantigens were loaded into DC-EV-based vaccines. This strategy elicited strong B-cell and T-cell responses and produced superior antitumor effects in preclinical models of melanoma and colorectal cancer [386].

**Fig. 6:**
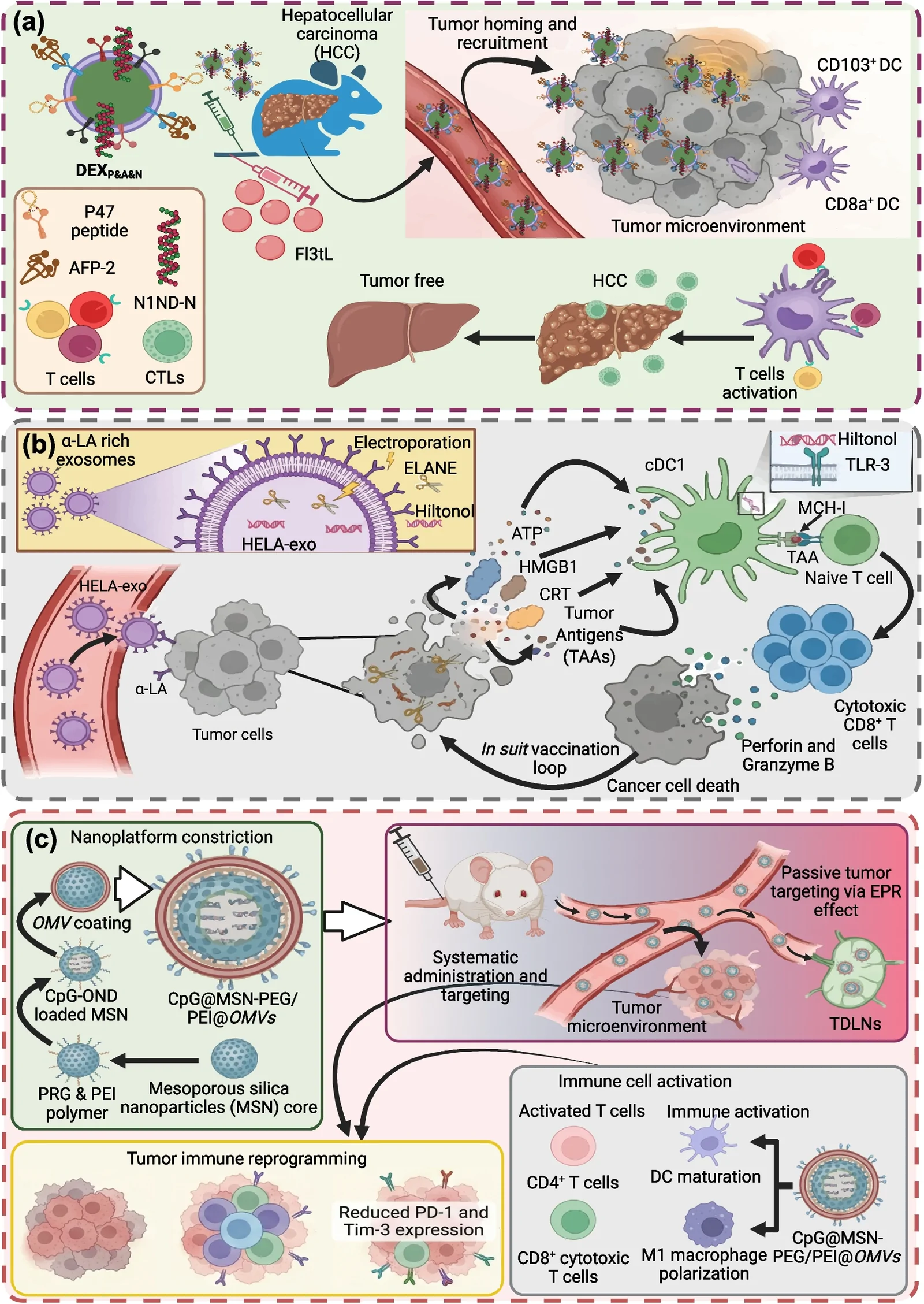
EVs in cancer vaccination and immunotherapy. **a** Mechanistic illustration of the antitumor immunogenicity of DC-EVs vaccines; DC-EVs are surface-modified using the CP05 anchor peptide to display three functional cargoes: the HCC-targeting peptide P47, the tumor antigen AFP, and the immunoadjuvant N1ND. Upon intravenous administration, the vaccine targets the tumor site via P47. The N1ND payload recruits and activates endogenous cross-presenting CD103^+^ DCs. These DCs uptake the vaccine and tumor neoantigens, leading to the cross-presentation of antigens to CD8^+^ T cells. This triggers a robust adaptive immune response and activates NK cells, resulting in tumor eradication and the formation of immunological memory. **b** Design and Mechanism of the HELA-Exo in situ vaccine. Illustration of HELA-Exos, engineered from allogenic breast cancer-derived exosomes, overexpress α-lactalbumin and are loaded with ELANE and Hiltonol to induce immunogenic cell death. These HELA-Exos activate DCs in situ within the tumor microenvironment, leading to the release of tumor antigens and DAMPs, which cross-prime tumor-specific CD8⁺ T cells. **c** Schematic illustration of CpG-loaded MSN-PEG/PEI nanoparticles are coated with bacterial outer membrane vesicles to form a biomimetic nanoplatform. After systemic administration, the nanoparticles accumulate in tumors and tumor-draining lymph nodes, where they are internalized by antigen-presenting cells. This process promotes DC maturation, macrophage activation, and T-cell–mediated immune responses, leading to enhanced cytokine production, reduced immunosuppressive signaling, and improved antitumor immunity. Figures were created in BioRender. https://BioRender.com/454y3zv

In another study [401], DC-EVs were engineered as a universal nano-vaccine for hepatocellular carcinoma by incorporating three functional components: a tumor-targeting peptide (P47-P), α-fetoprotein–derived antigens (AFP212-A2), and an immunoadjuvant domain that promoted DC recruitment and activation. This engineered DC-EVs vaccine (DEX_P&A2&N_) enhanced antigen cross-presentation and triggered de novo tumor-specific CD8⁺ T-cell responses, leading to significant tumor growth inhibition (Fig. 6a). Notably, when the peptide antigens were replaced with full-length α-fetoprotein, the vaccine achieved complete tumor eradication, and the inclusion of immune-supportive adjuvants further reinforced long-term antitumor memory. These findings demonstrate that DC-EV-based vaccines can coordinate innate and adaptive immune activation to achieve personalized antitumor immunity.

#### Tumor-derived EVs

TD-EVs typically reinforce cancer progression. They disseminate oncogenic proteins such as PD-L1, EGFR, and TGF-β, which suppress CD8^+^ T cell proliferation, promote apoptosis through PD-1 or Fas signaling, and stimulate the expansion of FOXP3^+^ regulatory T cells [402, 403]. Despite these immunosuppressive functions, TD-EVs naturally contain high concentrations of tumor antigens, making them attractive templates for vaccine engineering.

One strategy to harness TD-EVs for vaccination involves neutralizing their immunosuppressive components. For instance, silencing PD-L1 expression in TD-EVs using lentiviral-mediated shRNA enhanced DC activation and strengthened cytotoxic T-cell (CTL) responses [404]. A complementary approach circumvents intrinsic suppression by equipping TD-EVs with potent immunostimulatory adjuvants that override inhibitory signals. For example, a streptavidin–lactadherin fusion protein was used to anchor biotinylated CpG DNA onto TD-EV membranes, enabling simultaneous delivery of tumor antigens and TLR9 agonists to DCs and driving potent CD8⁺ T-cell activation [405]. In a recent study, TD-EVs derived from breast cancer cells were functionalized with α-lactalbumin and co-loaded with human neutrophil elastase (an inducer of immunogenic cell death) and the TLR3 agonist Hiltonol. This synergistic formulation selectively provoked ICD within the tumor microenvironment, leading to the release of tumor antigens under pro-inflammatory conditions. As a result, type 1 conventional dendritic cells (cDC1s) were effectively activated and enabled strong CD8⁺ T-cell priming and production that resulted in a significant tumor suppression in triple-negative breast cancer models (Fig. 6b) [406]. Furthermore, early-stage clinical studies using malignant ascites-derived EVs in colorectal cancer [407] have demonstrated acceptable safety profiles and preliminary immunogenicity, supporting further clinical development of TD-EV-based vaccines.

#### Other immune cell-derived EVs

EVs released from additional immune cell populations provide further opportunities to design modular immunotherapeutic platforms. EVs derived from M1-polarized macrophages contain proinflammatory mediators such as miR-155, IL-12, and inducible nitric oxide synthase (iNOS) that enable them to reprogram tumor-associated macrophages into an M1-like phenotype and strengthen antitumor immunity [408]. Experiments in breast cancer models demonstrated that these EVs significantly enhanced inflammatory cytokine production and promoted immune activation within the tumor microenvironment [409]. Building on this concept, hybrid EVs created by fusing membranes from M1 macrophages and tumor cells-termed artificial macrophage tumor exosomes (aMT-Exos)—have been developed. These hybrid vesicles retain the immunostimulatory properties of M1-EVs while gaining increased homing to lymph nodes and improved tumor accumulation, offering enhanced delivery specificity [410].

T cell-derived EVs introduce another level of immunological precision by carrying cytotoxic proteins such as perforin, granzyme B, and FAS ligand (FasL), which allow them to mediate direct tumor cell killing [411]. A major advancement in this category is the emergence of EVs released from CAR-T cells. These CAR-EVs retain tumor antigen specificity and cytotoxic potential but avoid severe toxicities, particularly cytokine release syndrome (CRS), that limit CAR T cell therapy, likely because, as acellular vehicles, they do not undergo the massive in vivo expansion that drives CRS [412, 413]. Similarly, NK cell-derived EVs exploit the natural cytotoxic machinery of innate immunity, including perforin, granzyme B, TRAIL, and FasL [414]. Beyond this direct cytotoxic activity, NK-EVs also deliver regulatory miRNAs. In neuroblastoma models, NK-EVs enriched with miR-186 targeted multiple oncogenic regulators such as MYCN and AURKA while also mitigating TGF-β-driven immune suppression, ultimately inhibiting tumor progression and cellular migration [157].

Collectively, these findings highlight the diverse immunological capabilities of engineered immune cell-derived EVs, emphasizing their potential to coordinate innate and adaptive immunity, improve antigen delivery, and remodel the tumor microenvironment.

#### Bacteria-outer membrane vesicles

Within cancer vaccination and immunotherapy, OMVs distinguish themselves from synthetic nanocarriers by serving as immunologically active bacterial entities with potent, intrinsic self-adjuvanting properties. This adjuvanticity, driven by pathogen-associated molecular patterns (PAMPs) such as LPS, enables OMVs to naturally bridge innate and adaptive immunity, making them versatile antigen delivery vehicles [415].

Exploiting this platform, engineered OMVs have demonstrated marked antitumor efficacy [416]. For instance, OMVs engineered to present the model tumor antigen ovalbumin (OVA) through a Cytolysin A (ClyA) fusion system effectively suppressed metastatic spread in a B16 melanoma model and demonstrated potent antitumor immunogenicity [124]. To address safety concerns, endotoxin-free *Clear Coli*-derived OMVs have been developed; when engineered to present the Neo-2/15 cytokine, these variants (CC-Neo2/15) significantly improved survival in colorectal cancer models compared to their unmodified counterparts [417]. Beyond direct tumor killing, OMVs have been integrated into hybrid nanoplatforms to remodel the tumor microenvironment (TME). In one study, OMVs cloaked onto CpG-loaded silica nanoparticles enabled efficient systemic tumor targeting. This formulation not only boosted intratumoral interferon-γ production and counteracted immunosuppressive TGF-β, but also promoted M1 macrophage polarization and durable T-cell memory. Notably, transcriptomic analyses revealed enhanced mitochondrial oxidative phosphorylation in T cells within tumor-draining lymph nodes, indicating alleviation of OMV-based strategies can metabolically reinvigorate exhausted T cells (Fig. 6c) [418].

Collectively, these findings establish OMVs as dual-function platforms that combine antigen delivery with microenvironment remodeling. However, successful clinical translation will necessitate precise titration of endotoxin bioactivity, antigen display density, and in vivo biodistribution, since uncontrolled inflammatory responses and off-target accumulation remain significant hurdles that demand a narrow therapeutic window.

### EVs-based vaccines in infectious diseases

EVs—including exosomes, microvesicles, and OMVs— represent a promising platform for vaccine development against a broad spectrum of infectious diseases, including COVID-19 [419], HIV/AIDS, tuberculosis (TB), and hepatitis B (HBV).

A major direction in EV-based vaccinology involves loading exogenous mRNA or siRNA into exosomes to enable transient antigen expression in target cells. In the context of SARS-CoV-2, one study employed electroporation to encapsulate mRNA encoding the viral spike protein into lung-derived exosomes. Following lyophilization, this formulation yielded a stable dry powder that maintained integrity for at least 28 days at room temperature. When administered via inhalation in African green monkeys, the mRNA-loaded EVs achieved uniform distribution across the respiratory tract and alveolar epithelium, eliciting robust systemic IgG and mucosal IgA responses [420]. Importantly, comparative analyses indicated that EVs exhibit negligible intrinsic immunogenicity even at high doses, positioning them as safer carriers compared to synthetic liposomes [421].

OMVs are another important infectious disease vaccine platform because they naturally display bacterial antigens and pathogen-associated molecular patterns that support self-adjuvanting immune activation. The clinical relevance of OMV-based vaccines is already well established, exemplified by the *Neisseria meningitidis* serogroup B vaccine 4CMenB (Bexsero), in which engineered OMVs present critical virulence-associated proteins [422]. OMV modularity has also been used to address antimicrobial-resistant bacterial infections. For example, bioengineered OMVs carrying the PcrV-HitA fusion antigen induced strong humoral and cellular immunity against *Pseudomonas aeruginosa*. In this design, bioengineered OMVs encapsulating the PcrV–HitA fusion antigen elicited strong humoral and cellular immunity in mice and provided cross-protection against several clinical isolates [423]. Similarly, Staphylococcus aureus OMVs displaying multiple virulence determinants generated functional antibody responses and protected animals in skin infection, sepsis, and renal abscess models [424]. For enteric disease, tetravalent OMVs derived from multiple Shigella serotypes induced systemic, mucosal, and cellular immune responses with broad cross-protection in murine models [425]. OMVs have also been explored for viral vaccines. In response to the SARS-CoV-2 pandemic, researchers engineered OMVs to display a mouse Cathelicidin-Related Antimicrobial Peptide (mCramp)–Spike fusion antigen. Immunization with these OMVs induced robust circulating IgG and mucosal IgA responses in hamsters, achieving viral neutralization and preventing pulmonary injury. These promising preclinical results supported progression to phase I clinical testing [426]. Earlier work demonstrated that OMVs presenting the conserved M2e epitope of Influenza A triggered strong cellular immunity and protected mice from lethal H1N1 challenge [427].

Regarding TB, which is caused by Mycobacterium tuberculosis (MTB) and still lacks a universally effective vaccine [428], EV-based vaccination has become an important area of innovations. Early evidence demonstrated that exosomes containing MTB antigens conferred meaningful protection against infection in mouse models [429]. Early evidence demonstrated the feasibility of EV-based acellular vaccines, prompting further optimization. A recent study combined immunoinformatics with EV biology to design an in silico multi-epitope peptide vaccine targeting MTB. Through systematic screening of EV-associated immunogenic proteins, researchers identified 35 high-affinity B-cell, helper T-cell (HTL), and CTL epitopes, excluding less suitable candidates such as GrpE. Researchers integrated these epitopes into a single construct and fused with the resuscitation-promoting factor E (RpfE) adjuvant to strengthen immune activation. Comprehensive structural validation confirmed that the engineered vaccine maintained a stable conformation and exhibited strong binding to the TLR4 receptor, with root-mean-square deviation (RMSD) values remaining below 0.44 nm. Moreover, in silico immune simulations predicted that this construct predicted to induce a durable protective response characterized by memory cell generation, elevated IFN-γ secretion, and robust IgM and IgG antibody production [430].

In the context of AIDS, where the progressive loss of CD4^+^ T lymphocytes drives disease severity [431], EVs offer diagnostic and therapeutic promise. In efforts to develop new prophylactic strategies against HIV-1, a recent study investigated a dual-component vaccine formulated from DC-EVs. Using lentiviral vectors, they engineered one population of exosomes to express the Nefmut-Tat fusion antigen, while a second population was designed to carry the adjuvant Hsp70. When administered together, this combined formulation demonstrated markedly greater immunogenicity compared with either component alone. The regimen induced a strong Th1-oriented immune profile, which is evidenced by elevated IgG2a antibody titers and robust production of IFN-γ, TNF-α, and Granzyme B. Importantly, this potent CTL response remained durable even after subsequent exposure to single-cycle replicating HIV-1, highlighting its promise as a prophylactic vaccine strategy [432]. This example also demonstrates that DC-EVs are not limited to cancer vaccination but can also be adapted for antiviral vaccine development.

Beyond HIV, EV-based strategies have also been explored for chronic hepatitis B, where immune exhaustion poses similar challenges. HBV, which causes chronic hepatitis in approximately 300 million individuals globally and significantly elevates the risk of cirrhosis and hepatocellular carcinoma [433, 434], has also benefited from EV research. One innovative therapeutic strategy utilizes an EVs-based vaccine derived from antigen-presenting cells (APCs). These APC-derived exosomes, primed with MHC I-specific peptides and immune activators like LPS, retain the activating properties of their parent APCs. Following intravenous administration, their small size and natural homing capabilities allow them to deliver antigens to immune organs (spleen) and target organs (liver), where they triggered targeted immune activation. This approach concurrently acts as an immunoregulator by facilitating the conversion of immunosuppressive M2 macrophages to pro-inflammatory M1 macrophages, thereby helping to overcome immune exhaustion and improve T-cell responsiveness in chronic HBV infection [435]. Similarly, another investigation revealed that EVs released by CD4^+^ T cells enhanced the humoral immune response elicited by hepatitis B surface antigen (HBsAg) vaccination, as reflected by elevated HBsAb levels in an antigen-specific manner [436], which confirms EVs' capacity to reinforce vaccine-induced immunity. Moreover, EV-based vaccines also show therapeutic potential for other pathogens. EVs released by macrophages infected with Salmonella act as effective antigen carriers. Intranasal administration of these EVs in mice activated mucosal immunity, stimulated Th1 cell expansion, and promoted the secretion of Th1 cytokines, including IFN-γ, alongside Salmonella-specific IgG antibodies [437]. Collectively, these findings highlight the versatility of EVs from different sources as modular platforms for both prophylactic and therapeutic vaccination across cancer, infection, or other diseases. Table 4 summarizes representative EV-based vaccine studies categorized by EV source, engineering strategies, target indications, and key immunological outcomes.

**Table 4:** EVs-based platforms for cancer immunotherapy and infectious disease vaccination

Platform/EV type	Representative engineering strategy	Target indication(s)	Key immunological/therapeutic outcomes	Quantitative/therapeutic outcome	Reference
NK cell-EVs	NK-EVs enriched with miR-186	Neuroblastoma	Targeted MYCN, AURKA, and TGF-β signaling, inhibited tumor progression and migration, and preserved NK-associated cytotoxic activity in a cell-free format	Median survival increased to 60 days vs 45–52 days in controls	[157]
DC-EVs	DC-EVs presenting TAAs on MHC I/II with immune-stimulatory molecules	Preclinical tumor and CML models	Activated NK cells and promoted CD4⁺/CD8⁺ T-cell antitumor responses, supporting coordinated innate and adaptive immunity	Suppressed established tumors and prolonged survival in CML mouse models	[395–397]
Antigen-optimized DC-EVs	DC-EVs loaded with full-length antigen or patient-specific neoantigens	Melanoma, colorectal cancer	Enhanced antigen-specific CD4⁺/CD8⁺ T-cell responses, promoted B-cell activation, and improved antitumor immunity compared with simpler antigen formulations	Increased survival and eliminated lung metastasis in B16F10 models	[386, 399]
TD-EVs with reduced suppression	TD-EVs with PD-L1 silencing	Acute lymphocytic leukemia	Reduced exosomal PD-L1-mediated immunosuppression, improved DC maturation, enhanced T-cell activation, and strengthened anti-leukemia CTL responses	MST increased to 31–32 days; tumor growth delay reached 50.1 ± 4.1%	[404]
TD-EVs-based in situ vaccination	α-lactalbumin-functionalized TD-EVs co-delivering ELANE and Hiltonol	Triple-negative breast cancer	Induced immunogenic cell death, promoted tumor antigen release, activated cDC1s in situ, and enhanced tumor-reactive CD8⁺ T-cell priming	HELA-Exos showed the lowest tumor volume and strongest tumor inhibition	[406]
Macrophage EVs	M1 macrophage EVs or IL4R-targeted M1-EVs loaded with immune-regulatory cargo	Breast cancer models	Reprogrammed tumor-associated macrophages toward an M1-like phenotype, increased pro-inflammatory antitumor signaling, and enhanced immune-mediated tumor suppression	PTX-M1-EVs showed stronger tumor inhibition than PTX or M1-EVs alone	[408, 409]
Hybrid macrophage-tumor EVs	Artificial macrophage–tumor EVs from fused M1 and tumor membranes	Solid tumors	Combined M1 macrophage-derived immune stimulation with tumor membrane-derived homing properties, improving lymph-node targeting and tumor accumulation	-	[410]
T cell and CAR-T cell EVs	EVs carrying perforin/granzyme B; CAR-EVs retaining CAR specificity	Various cancers	Mediated direct antigen-specific tumor-cell killing through cytotoxic molecules while retaining CAR-like targeting specificity and reducing systemic toxicity risks	10 μg CAR-EVs caused approximately 20% tumor-cell killing and showed lower CRS-like toxicity than CAR-T cells	[411, 413]
OMVs for cancer immunotherapy	OMVs presenting OVA via ClyA or endotoxin-reduced OMVs displaying Neo-2/15	Melanoma, colorectal cancer	Activated DC maturation, promoted tumor-specific cytotoxic T-cell immunity, enhanced tumor rejection, and improved OMV safety through endotoxin reduction	ClyA-OVA OMVs nearly eliminated B16-OVA lung metastases; LPS removal increased maximum tolerated dose by > 25-fold	[124, 417]
EV-based inhalable COVID-19 vaccines	Lung-derived EVs displaying SARS-CoV-2 RBD or encapsulating spike mRNA	COVID-19	Distributed efficiently in the respiratory tract and induced systemic IgG, mucosal IgA, and lung T-cell responses after inhalation delivery	RBD-EVs cleared pseudovirus in mice and reduced pneumonia in hamsters; spike mRNA EVs showed stronger IgG/SIgA than liposomes	[420]
OMVs against AMR bacteria	Bioengineered OMVs carrying PcrV–HitA or multiple *S. aureus* virulence antigens	*Pseudomonas* and *Staphylococcus* infections	Induced strong humoral and Th1/Th17 cellular responses, promoted functional antibacterial antibodies, and protected against multiple bacterial infection models	OMV-PH gave 70% protection against lethal *P. aeruginosa* challenge; CLSH-OMVs gave 100% protection in a sepsis model	[423, 424]
OMVs against enteric pathogens	Tetravalent OMVs from multiple *Shigella* serotypes	Shigellosis	Generated systemic, mucosal, and cellular immune responses with broad cross-protection against clinically relevant dysentery-causing *Shigella* strains	Immunized mice showed significantly higher survival and lower intestinal/liver bacterial burden after challenge	[425]
OMV-based viral vaccines	OMVs displaying mCramp–Spike fusion antigen or conserved M2e epitope	COVID-19, H1N1 influenza	Induced systemic and mucosal antiviral antibody responses, promoted cellular immunity, and protected animals from viral challenge-associated disease	OMV-mC-Spike prevented lung lesions after SARS-CoV-2 challenge; M2e4xHet-OMVs gave 100% survival after lethal influenza challenge	[426, 427]
EV vaccines for tuberculosis	EVs carrying *M. tuberculosis* antigens	Tuberculosis	Induced antigen-specific Th1-type immunity with IFN-γ/IL-2-producing T cells and protected against *M. tuberculosis* infection	CFP-EVs gave protection comparable to BCG and ~0.5-log better lung protection than BCG	[429]
EV-based HIV prophylactic vaccines	DC-EVs carrying Nefmut–Tat antigen plus Hsp70-EVs	HIV-1	Induced Th1-biased humoral immunity and CTL-associated responses, including IgG2a, IFN-γ, TNF-α, and granzyme B production	Increased IgG2a, IFN-γ, TNF-α, and granzyme B after HIV-1 antigen exposure	[432]
EVs enhancing HBV vaccination	CD4⁺ T-cell EVs combined with HBsAg vaccine	Hepatitis B vaccination	Enhanced B-cell activation, proliferation, plasma-cell formation, and antigen-specific humoral responses to HBsAg vaccination	Increased HBsAb IgG/IgG2a levels and bone marrow plasma cells	[436]
EV vaccines for *Salmonella*	EVs from *Salmonella*-infected macrophages	*Salmonella* infection	Activated mucosal immunity, expanded Th1-type CD4⁺ T-cell responses, increased IFN-γ production, and induced *Salmonella*-specific IgG antibodies	40 μg EVs produced antibody titers comparable to live ΔaroA vaccine	[437]

### EVs in clinical trials

Advancements in research and technology are driving EV-based therapies toward becoming a significant aspect of modern medicine. The first clinical trials investigating EVs, such as DC-EVs loaded with tumor antigens, which naturally carry MHC molecules, were first explored EVs' potential in cancer vaccination. In addition, MSC-EVs are gaining attention in clinical trials for their regenerative and immunomodulatory properties. EV-based therapeutics are being currently investigated in various cancers, as well as inflammatory and neurological disorders. Clinical research in this field is particularly active in the United States and China. Table 5 highlights the clinical translation status of EV-based interventions, including trial phase, route of administration, sponsor, and registration number.

**Table 5:** Selected registered clinical trials of EVs based therapeutics (https://www.clinicaltrials.gov/)

No	Condition	Specific EV product/name	EV source/type and route	Clinical phase/status	Sponsor	Registration No
1	Moderate-to-severe ARDS	ExoFlo	BMSC-EVs; intravenous administration versus placebo/normal saline	Phase 3; recruiting; multicenter randomized double-blind placebo-controlled trial	Direct Biologics, LLC	NCT05354141
2	Perianal fistulizing Crohn’s disease	ExoFlo	Ex vivo culture-expanded adult allogeneic BMSC-EVs isolate; local injection versus normal saline	Phase IB/IIA; terminated due to enrollment challenges; randomized, single-blind, placebo-controlled dose-escalation trial	Direct Biologics, LLC	NCT05836883
3	Medically refractory Crohn’s disease	ExoFlo	Ex vivo culture-expanded adult allogeneic BMSC-EVs isolate; intravenous administration	Phase 1; terminated due to enrollment challenges; open-label multicenter safety study with exploratory efficacy assessment	Direct Biologics, LLC	NCT05130983
4	Medically refractory ulcerative colitis	ExoFlo	Ex vivo culture-expanded adult allogeneic BMSC-EVs isolate; intravenous administration	Phase 1; terminated due to enrollment challenges; open-label multicenter safety study with exploratory efficacy assessment	Direct Biologics, LLC	NCT05176366
5	Dystrophic epidermolysis bullosa wounds	AGLE-102	Allogeneic normal donor MSC-EV product; local wound application with standard of care versus standard of care alone	Phase 1/2A; recruiting; randomized multicenter open-label study with intra-subject paired wound control	Aegle Therapeutics	NCT04173650
6	Deep second-degree burn wounds	Not specified	BMSC-EVs; local wound administration	Phase 1; non-recruiting; safety and preliminary efficacy study	Aegle Therapeutics	NCT05078385
7	Moderate-to-severe atopic dermatitis	BxC-I17e	Primed iMSC-EVs; subcutaneous single and multiple ascending-dose injection versus placebo	Phase 1; recruiting; randomized double-blind placebo-controlled study	Brexogen Inc	NCT06055361
8	Healthy participants	ILB-202	Engineered exosome loaded with super-repressor IκBα; intravenous single ascending-dose administration versus placebo	Phase 1; completed; single-center randomized double-blind placebo-controlled safety and tolerability study	ILIAS Biologics Inc	NCT05843799
9	ARDS or novel coronavirus pneumonia caused by COVID-19	Not specified	MSCs exosomes; intravenous injection	Phase 1/2; active, not recruiting; safety and efficacy study in moderate-to-severe ARDS/NCP	AVEM HealthCare	NCT04798716
10	Diabetic foot ulcers	PEP-TISSEEL	Purified exosome product (PEP) with TISSEEL; topical application plus standard of care versus standard of care alone	Phase 2a; completed; multicenter prospective randomized controlled safety and efficacy study	Rion Inc	NCT06319287
11	Knee osteoarthritis	PEP with or without EUFLEXXA	PEP reconstituted with 0.9% normal saline; single intra-articular injection at low or high dose, with or without EUFLEXXA	Phase 1b; recruitment status not specified in shared record; safety and exploratory efficacy study	Rion Inc	NCT06463132
12	Bronchopulmonary dysplasia in extremely preterm infants	EXOB-001	Allogeneic UC-MSC-EVs; intratracheal administration via endotracheal tube versus saline placebo	Phase 1/2; recruiting; phase I dose-escalation followed by phase II randomized placebo-controlled dose-finding trial	EXO Biologics S.A	NCT06279741
13	Acute ischemic stroke	GD-iExo-003	Human iMSCs exosomes; intravenous administration versus exosome placebo	Phase 1/2; recruiting; multicenter randomized double-blind placebo-controlled dose-escalation and expanded safety study	Xuanwu Hospital, Beijing	NCT06138210
14	Recurrent or metastatic bladder/urothelial cancer	Personalized chimeric exosomal tumor vaccine	Patient-specific chimeric exosome vaccine prepared from tumor cells and peripheral blood DCs or macrophages; route not specified in shared record	Phase 1; active/recruiting; open-label dose-escalation safety and tolerability study	Shanghai Pudong Hospital	NCT05559177
15	Severe COVID-19 viral pneumonia/respiratory failure	XoGlo®	Purified placental-MSCs exosomes; intravenous infusion versus saline placebo	Phase 2; completed/no longer recruiting; randomized parallel trial	Kimera Labs	ISRCTN33578935
16	Unresectable non-small-cell lung cancer responding or stable after induction chemotherapy	Dex2	Tumor antigen-loaded DCs exosomes; intradermal injections following metronomic cyclophosphamide	Phase 2; completed; open-label single-group maintenance immunotherapy trial	Gustave Roussy, Cancer Campus, Grand Paris	NCT01159288
17	Healthy volunteers/lung safety evaluation	MSCs-Exo	Allogenic ADMSCs exosomes; aerosol inhalation/nebulization	Phase 1; open-label, non-randomized tolerance study with escalating nebulized doses	Ruijin Hospital, Shanghai Jiao Tong University School of Medicine	NCT04313647
18	Metastatic pancreatic ductal adenocarcinoma with KRASG12D mutation	iExosomes	MSCs exosomes loaded with KRASG12D siRNA; intravenous infusion	Phase 1/2; recruiting; open-label single-arm dose-escalation study assessing MTD/DLT and preliminary efficacy	M.D. Anderson Cancer Center	NCT03608631
19	Moderate/severe COVID-19 infection	EXO-CD24	T-REx™−293 cell exosomes engineered to overexpress CD24; aerosolized inhalation as add-on to standard care	Phase 1; unknown status, last known recruiting; open-label dose-escalation safety study	Tel-Aviv Sourasky Medical Center	NCT04747574
20	Moderate/severe COVID-19 infection	CD24-overexpressing exosomes	Exosomes overexpressing CD24; mouthpiece nebulization/inhalation once daily for 5 days; two-dose comparison as add-on to standard care	Phase 2; active/recruiting; randomized single-blind dose-comparison safety and efficacy study	Athens Medical Society	NCT04902183
21	Severe COVID-19-related pneumonia	haMSC-Exos	Human allogenic ADMSC exosomes; aerosol inhalation/nebulization once daily for 5 days	Phase 2a; completed pilot study; single-arm open-label interventional trial	Jinyintan Hospital/Ruijin Hospital, Shanghai Jiao Tong University School of Medicine	NCT04276987
22	COVID-19-associated two-sided pneumonia	EXO 1/EXO 2 inhalation	Exosomes; aerosol inhalation versus placebo inhalation	Phase 2; unknown status, last known enrolling by invitation; safety and efficacy study	Olga Tyumina	NCT04602442
23	Oral mucositis associated with chemoradiation for head and neck cancer	Grape extract/grape exosomes	Grape exosomes administered orally as grape extract/powder during chemoradiation versus standard oral mucositis therapy	Phase 1; completed; randomized open-label preliminary clinical trial	University of Louisville	NCT01668849
24	Inflammatory bowel disease	Ginger exosomes ± curcumin	Ginger exosomes alone or combined with curcumin; oral administration daily for 28 days	Not applicable; completed; randomized open-label exploratory trial	University of Louisville	NCT04879810
25	Colon cancer	Curcumin conjugated with plant EVs	PDEVs mediated oral delivery of curcumin to normal and malignant colon tissue; compared with curcumin alone and no treatment	Not applicable; unknown status, last known recruiting; randomized open-label basic-science trial	University of Louisville	NCT01294072

Despite this expanding clinical pipeline, a substantial gap remains between promising preclinical findings and established clinical efficacy. Most EV therapeutics remain in early-phase development, primarily assessing safety, tolerability, dose selection, and preliminary efficacy, whereas relatively few products have progressed to larger confirmatory trials [21]. As shown in Table 5, the available evidence is further limited by incomplete or unavailable results, uncertain recruitment status, and the termination of some studies because of enrollment difficulties as well as the practical challenges of production costs and dosage. Differences in EV source, donor characteristics, product composition, manufacturing process, dose, administration route, comparators, and outcome measures also hinder direct comparison and replication across studies. Consequently, standardized manufacturing and characterization procedures and validated potency assays are required to establish consistent product quality and biological activity [211]. Thus, current trials demonstrate the feasibility and broad therapeutic potential of EV-based interventions but do not yet provide sufficient evidence of reproducible efficacy, optimal dosing, or long-term safety to support widespread clinical implementation.

## Challenges for EVs as next-gen therapeutics and drug delivery platforms

EVs emerged as therapeutic agents and a versatile natural carrier for drug delivery. Earlier sections of this review discussed their biogenesis, sources, isolation methods, engineering strategies, and applications. Despite the progress in EV research, the clinical translation of EV-based therapeutics is still limited by several unresolved challenges. These challenges include issues related to scalable and standardized production, cargo loading and engineering, product characterization and stability, vaccine-specific hurdles, and safety and immunogenicity. Here, we summarize the key challenges and highlight aspects that must be addressed to enable reliable and clinically applicable EV-based therapies.

### Scalability, standardization, isolation, and purification-related challenges

Despite rapid progress in EV-based therapeutics, clinical translation remains limited by the lack of scalable and standardized workflows that can maintain EV purity, identity, potency, and batch-to-batch consistency. EV yield and composition are strongly affected by upstream variables such as producer-cell source, donor background, passage number, physiological state, culture medium, stimulation conditions, and harvesting time. These factors can alter vesicle concentration, cargo composition, and biological activity, thereby complicating quality control and regulatory approval [438]. For bacterial OMVs, scalable fermentation is easier, but purification must control strain-dependent heterogeneity, growth-phase variation, endotoxin levels, and toxin-associated contaminants.

PDEVs present a different scalability profile. They can be produced from abundant and low-cost plant materials, but their standardization is complicated by plant species, cultivar, growth conditions, ripening stage, harvest time, storage, and extraction method [439]. Their isolation also differs from mammalian EV isolation because rigid plant cell walls often require mechanical, enzymatic, or chemical disruption before vesicle enrichment. This can increase co-isolation of plant proteins, polysaccharides, pigments, cell wall fragments, secondary metabolites, and protein aggregates. In addition, universal plant EV markers and subtype definitions remain insufficiently established, making identity testing, marker-based purification, and cross-study comparison difficult [128].

EV heterogeneity further complicates purification and standardization across all EV platforms. EV populations may contain vesicles with different biogenetic origins, sizes, membrane compositions, surface markers, and bioactive cargos. These subpopulations can differ in uptake, biodistribution, potency, and safety. Currently, EV isolation methods include ultracentrifugation-based techniques, size-based approaches, immunoaffinity capture, precipitation, and microfluidic platforms. Each method has distinct trade-offs in yield, purity, cost, scalability, and preservation of EV bioactivity [228]. These methods may also co-isolate non-vesicular contaminants, such as lipoproteins, protein aggregates, free nucleic acids, residual culture components, or plant-derived impurities depending on the source [440]. Therefore, no single method is sufficient for all EV products.

TFF is one of the more practical options for large-scale EV production because it is compatible with closed, high-throughput, and Good Manufacturing Practice (GMP)-oriented systems. However, it often needs additional purification steps such as density-gradient ultracentrifugation, SEC, or affinity-based purification to remove non-vesicular contaminants [438]. In our view, the most realistic path toward solving the scalability and standardization problem is not the search for a single perfect method but the rational integration of a limited number of robust complementary techniques into platform-specific workflow systems, which is supported by head-to-head method comparisons, clearly defined critical process parameters, validated potency assays, and quality-control criteria capable of ensuring batch consistency and satisfying regulatory requirements.

### Engineering, cargo loading, and targeting reproducibility-related challenges

EV engineering can improve therapeutic performance by enabling cargo loading, surface modification, targeted delivery, and controlled pharmacokinetics. However, reproducible engineering remains difficult. A major limitation is low loading capacity because EVs already contain endogenous proteins, RNAs, lipids, and metabolites. Reported loading efficiencies are about 20–30% for small molecules, less than 2% for nucleic acids, and around 20% for proteins [130]. Pushing higher loading often needs sonication, electroporation, freeze–thaw cycles, or repeated processing, which can damage EV membranes and disturb native cargo. Conversely, low loading efficacy necessitates higher doses of EVs, which may complicate production and safety evaluation. Low encapsulation also creates purification problems. After engineering large amount of free cargo often remains. It must be removed to avoid toxicity and to know the true drug content per vesicle. Multiple washing or polishing steps are usually required, which may reduce yield, extend process time, and can affect sterility. Complete removal of free cargo is rarely achieved. Protocols and characterization depth also differ widely between studies. In this context, the lack of specific consensus criteria for engineered EVs is a major limitation, and dedicated recommendations similar to the MISEV 2018 and 2023 frameworks for naïve vesicles [41, 441] are needed to standardize reporting of loading metrics, acceptable residual cargo levels, encapsulation efficacy, and minimal physicochemical and biochemical characterization for engineered EVs.

These engineering challenges also differ between plant and non-plant EVs. For mammalian EVs, endogenous loading often requires large-scale donor-cell manipulation, such as transfection or genetic engineering, followed by removal of transfection reagents and culture-related impurities [440]. OMVs are easier to engineer genetically, but their modification must be balanced with strict control of inflammatory PAMPs, endotoxin activity, and toxin-associated cargo. In contrast, PDEVs are attractive for oral delivery and natural bioactive cargo transport, but their engineering is less standardized. Their plant-specific lipid, polysaccharide, protein, small RNA, and phytochemical composition may influence loading efficiency, surface modification, targeting behavior, and batch reproducibility [128].

Targeting reproducibility is another major challenge. Surface engineering does not generate a fully uniform EV population because individual vesicles may display different ligand densities, orientations, and accessibility. Ligands may also degrade, detach, or become masked by protein corona formation after administration, which can change EV uptake, clearance, and biodistribution [298]. In addition, receptor expression varies among patients, disease stages, tissues, and cell subpopulations. Therefore, targeted EV products require quantitative release criteria for ligand density, receptor binding, target-cell uptake, off-target uptake, biodistribution, and functional potency [21]. Finally, engineered EVs require optimized storage and stability protocols. Mechanical and chemical engineering steps may promote aggregation, membrane disruption, ligand loss, or altered release behavior. It is still unclear whether storage conditions used for native EVs, such as –80 °C, are suitable for engineered EVs carrying sensitive drugs, RNAs, or proteins [130]. Freeze–thaw cycles may cause aggregation, loss of surface ligands, or changes in release behavior. Systematic studies of storage temperature, duration, freeze–thaw cycles, and lyophilization are needed. Only then can robust protocols be set to preserve the structure, loading efficiency, and biological activity of engineered EVs for clinical use.

### Regulatory-related challenges

Regulatory uncertainty remains a major barrier to the clinical translation of EV-based therapeutics. As we earlier discussed, EVs are complex biological products composed of heterogeneous vesicle populations containing proteins, lipids, nucleic acids, and surface molecules derived from their parental cells. Their regulatory classification depends on the EV source, manufacturing process, degree of modification, incorporated cargo, intended use, and proposed mechanism of action. A central difficulty is that the active substance of an EV product may not be a single defined molecule. Instead, therapeutic activity may result from membrane components, vesicular cargo, or combined EV-associated signals. This makes mechanism-of-action definition and potency assay development difficult during early product development [442].

These regulatory challenges are also source dependent. For non-plant EVs, especially mammalian EVs, regulators must consider producer-cell qualification, donor screening, cell-bank consistency, culture conditions, adventitious-agent testing, and risks linked to oncogenic, immunogenic, or procoagulant cargo. For bacterial OMVs, endotoxin content, toxin-associated components, inflammatory potency, and strain-specific safety must be tightly controlled. In contrast, plant-derived EVs face additional classification challenges because they may fall between food-derived products, natural products, biologics, and nanomedicines. Their active components may include plant lipids, proteins, RNAs, polysaccharides, and phytochemicals, making potency definition and quality control more complex. Plant source, cultivation conditions, pesticide or agrochemical residues, extraction method, and batch consistency must also be considered during regulatory evaluation [439].

A reproducible EV drug product requires well-defined manufacturing documentation. This should include source material, raw materials, isolation and purification methods, formulation, storage conditions, in-process controls, and release criteria. Critical quality attributes should include particle concentration, size distribution, morphology, membrane integrity, surface-marker profile, cargo composition, sterility, endotoxin level, mycoplasma absence, and freedom from adventitious agents or process-related impurities [443]. MISEV2023 also emphasizes transparent reporting of EV source, separation, characterization, storage, and functional attribution, which is essential for reproducibility and regulatory evaluation [41]. For clinical-grade EV manufacturing, the principle that “the process is the product” is particularly important because process changes can directly influence final EV composition and activity [444].

Potency testing remains one of the most difficult regulatory requirements. Particle number, total protein content, or a few surface markers alone cannot predict therapeutic activity. Potency assays should instead be linked to the intended mechanism of action and should include functional readouts such as immunomodulation, angiogenesis, anti-apoptotic activity, tissue repair, or target-cell recovery [445]. For engineered EVs, additional requirements include evaluation of loading efficiency, residual free cargo, residual processing reagents, cargo stability, surface-modification reproducibility, and potential changes in biodistribution or off-target uptake [446]. Therefore, successful regulatory translation of EV therapeutics requires product-specific release criteria, validated potency assays, robust comparability testing, long-term safety monitoring, and early interaction with regulatory authorities.

### EVs as vaccine-related challenges

EV-based vaccines offer a versatile platform for delivering tumor or pathogen antigens together with endogenous damage-associated molecular patterns (DAMPs). However, their clinical translation faces several vaccine-specific challenges that go beyond general issues of EV, such as isolation or cargo loading. A key problem is the choice of producer cells. DC-EVs are attractive because they display peptide-loaded MHC, costimulatory molecules, and NK-activating ligands. Clinical-grade DC-EVs have been evaluated in early clinical studies, showing acceptable safety [447]. They generally lack a strong immunosuppressive cargo. Even so, their yield is low, and the cost of generating large numbers of functional DCs remains high, which limits broad clinical use [12]. Alternative sources, such as tumor cells or immortalized epithelial lines, can provide higher EV output. Yet, TD-EVs may carry oncogenic or immunosuppressive signals, and immortalized epithelial cells raise concerns about genomic instability and long-term safety [11]. This makes it difficult to define a single safe and effective vaccine platform and complicates regulatory assessment of source choice for prophylactic or therapeutic vaccination.

Another key challenge is the control of antigen presentation and immune activation. EV vaccines can be engineered through endogenous or exogenous antigen-loading strategies, but these approaches may differ in antigen localization, density, orientation, stability, and accessibility to immune cells. APC-derived EVs can incorporate MHC-peptide complexes together with co-stimulatory molecules such as CD86 and ICAM-1, allowing them to function as immunologically active antigen-presenting vesicles [448]. However, for clinical use, it is not sufficient to confirm antigen loading alone; it is also necessary to define whether the antigen is surface-displayed, luminally encapsulated, associated with MHC-peptide complexes, or released for processing by recipient antigen-presenting cells. This distinction is important because surface-displayed peptide-MHC complexes may directly engage T cells, whereas luminal antigens depend on uptake, processing, and presentation by recipient APCs. Similarly, the balance between co-stimulatory molecules and inhibitory signals determines whether EVs promote productive T-cell priming, immune tolerance, or excessive inflammation. Poorly controlled antigen loading may result in weak T-cell priming and inconsistent therapeutic efficacy, whereas excessive or unstable antigen presentation may cause unpredictable immune activation. Therefore, antigen identity, antigen density, MHC-peptide presentation, co-stimulatory or inhibitory signals, and the magnitude of immune activation must be carefully controlled.

Moreover, EV vaccines are constrained by biological heterogeneity and complex behavior in vivo. EVs contain diverse proteins, lipids, and nucleic acids that reflect the state of their donor cells. This complexity can be advantageous for multivalent immune stimulation, but it also makes it hard to control which signals dominate the response. After systemic administration, EVs are further remodeled by interactions with blood components. A protein corona can rapidly form on the vesicle surface, masking engineered ligands or antigenic epitopes and altering uptake by antigen-presenting cells [298]. This process may blunt antigen presentation or nucleic acid delivery and can shift biodistribution toward clearance organs. Overall, successful EV vaccine development will require careful balancing of immune-stimulation and safety, rational selection of producer cells, and a better systems-level understanding of how EVs are remodeled and trafficked in vivo rather than relying solely on their engineered features at the time of administration. In parallel, standardized vaccine-specific potency assays will be needed to confirm reproducible antigen presentation, APC maturation, CD4⁺ and CD8⁺ T-cell activation, NK-cell stimulation, cytokine profiles, and absence of unwanted immunosuppressive or hyperinflammatory responses before clinical application.

### Safety and immunogenicity-related challenges

EVs are often described as safer and less immunogenic than cell therapies. This makes them attractive for repeated or long-term use. However, their safety and immunogenicity are not uniform. They depend strongly on the cellular source, production conditions, and route and frequency of administration [449]. Autologous mammalian EVs generally have a lower risk of immune rejection, but they are difficult to standardize and scale. Allogeneic EVs from healthy donors or standardized cell lines are easier to manufacture, but they may trigger innate or adaptive immune responses, antibody formation, or altered clearance after repeated dosing. This requires strict donor screening, robust pathogen testing, and careful monitoring of proinflammatory, procoagulant, or oncogenic cargo. From a regulatory perspective, allogeneic products can follow more established biologics pathways but must show high consistency and a clear safety margin for chronic use. Future approaches, such as using hypoimmunogenic or gene-edited universal donor cells, may reduce these risks and support broader clinical application of EV-based therapies.

Safety concerns also differ among non-plant EV sources. TD-EVs may contain oncogenic proteins, immunosuppressive molecules, or metastasis-promoting signals, so their use requires careful removal or neutralization of harmful cargo. Immune-cell-derived EVs may induce excessive immune activation if antigen presentation, cytokine-associated cargo, or co-stimulatory signals are not controlled. OMVs have strong immunostimulatory potential, but their safety is limited by LPS, toxins, and other inflammatory PAMPs. Endotoxin reduction, strain engineering, and dose optimization are therefore essential for OMV-based products. PDEVs usually show favorable biocompatibility and low toxicity in preclinical studies, especially for oral administration. However, their safety challenges are different from mammalian and bacterial EVs. Potential concerns include plant allergenicity, pesticide or agrochemical residues, co-isolated plant metabolites, and unclear long-term biodistribution after repeated oral or systemic exposure. In addition, the biological activity of plant RNAs, lipids, proteins, and phytochemicals may vary between plant sources and batches, making chronic safety evaluation important. Overall, EV safety assessment should be tailored to the EV source and intended use. Future studies should evaluate acute and chronic toxicity, biodistribution, immune activation, thrombogenicity, organ accumulation, genotoxic or tumor-promoting risks, and repeated-dose safety before EV-based therapeutics can be widely translated into clinical practice.

## Conclusion

EVs are emerging as biologically grounded platforms for next-gen therapeutics because their natural functions can be translated into therapeutic design. Their ability to protect bioactive cargo, interact with recipient cells, cross biological barriers, and retain source-specific molecular signatures gives them value beyond conventional nanocarriers. In this sense, EVs provide a bridge between endogenous intercellular communication and engineered delivery systems, offering opportunities for drug delivery, immune modulation, cancer therapy, vaccination, and tissue repair.

The therapeutic promise of EVs depends strongly on matching the right vesicle source with the right clinical purpose. Mammalian cell-derived EVs, PDEVs, and bacteria-EVs differ in composition, biological activity, manufacturability, safety, and clinical readiness. These differences should be viewed as design options rather than simple limitations. By selecting appropriate EV sources and combining them with suitable isolation and engineering strategies, EV platforms can be adapted for diverse disease models and translational needs. Looking ahead, the field must move from proof-of-concept studies toward rational, standardized, and clinically reliable EV products. This requires integrated progress in EV biology, scalable manufacturing, quality control, cargo loading, targeting, safety evaluation, and regulatory alignment. If these elements are developed in parallel, EVs may become a versatile therapeutic toolkit for precision nanomedicine, personalized treatment, vaccines, combination therapies, and regenerative medicine. Ultimately, the success of EV-based therapeutics will depend not only on their natural advantages, but also on how effectively these biological properties are converted into reproducible and clinically meaningful interventions.

## Data Availability

Not applicable. This manuscript is a review article and does not involve the generation or analysis of new experimental data.
